# Sensitive bioluminescence imaging of fungal dissemination to the brain in mouse models of cryptococcosis

**DOI:** 10.1242/dmm.039123

**Published:** 2019-06-17

**Authors:** Liesbeth Vanherp, Alexandra Ristani, Jennifer Poelmans, Amy Hillen, Katrien Lagrou, Guilhem Janbon, Matthias Brock, Uwe Himmelreich, Greetje Vande Velde

**Affiliations:** 1 Biomedical MRI, Department of Imaging and Pathology, KU Leuven, 3000 Leuven, Belgium; 2 Molecular Small Animal Imaging Center (MoSAIC), KU Leuven, 3000 Leuven, Belgium; 3 Laboratory of Clinical Bacteriology and Mycology, Department of Microbiology and Immunology, KU Leuven, 3000 Leuven, Belgium; 4 RNA Biology of Fungal Pathogens, Department of Mycology, Pasteur Institute, Paris 75015, France; 5 Fungal Biology Group, School of Life Sciences, University of Nottingham, Nottingham NG7 2RD, UK

**Keywords:** *Cryptococcus*, Pulmonary disease, Mouse model, Fungal infection, Blood-brain barrier, Non-invasive imaging

## Abstract

*Cryptococcus neoformans* is a leading cause of fungal brain infection, but the mechanism of dissemination and dynamics of cerebral infection following pulmonary disease are poorly understood. To address these questions, non-invasive techniques that can study the dynamic processes of disease development and progression in living animal models or patients are required. As such, bioluminescence imaging (BLI) has emerged as a powerful tool to evaluate the spatial and temporal distribution of infection in living animals. We aimed to study the time profile of the dissemination of cryptococcosis from the lung to the brain in murine models by engineering the first bioluminescent *C. neoformans* KN99α strain, expressing a sequence-optimized red-shifted luciferase. The high pathogen specificity and sensitivity of BLI was complemented by the three-dimensional anatomical information from micro-computed tomography (μCT) of the lung and magnetic resonance imaging (MRI) of the brain. These non-invasive imaging techniques provided longitudinal readouts on the spatial and temporal distribution of infection following intravenous, intranasal or endotracheal routes of inoculation. Furthermore, the imaging results correlated strongly with the fungal load in the respective organs. By obtaining dynamic and quantitative information about the extent and timing of brain infections for individual animals, we found that dissemination to the brain after primary infection of the lung is likely a late-stage event with a timeframe that is variable between animals. This novel tool in *Cryptococcus* research can aid the identification of host and pathogen factors involved in this process, and supports development of novel preventive or therapeutic approaches.

## Abstract (precis)

**Summary:** A novel combination of bioluminescence and anatomical imaging non-invasively identified the timeframe and extent of *Cryptococcus neoformans* dissemination to the brain in animal models of systemic and pulmonary fungal infection.

## INTRODUCTION

*Cryptococcus neoformans* is an important pathogen to immunocompromised individuals, particularly HIV patients. This yeast is the most common cause of fungal infections in the central nervous system, and prompt initiation of appropriate treatment is crucial to patient survival (Schwartz et al., 2018). Infection can start upon inhalation of *Cryptococcus* cells present in the environment. After a pulmonary infection or after reactivation of dormant disease, susceptible individuals can develop a disseminated infection with a predilection to the brain (Lin and Heitman, 2006).

The exact mechanism and the dynamics of *C. neoformans* disseminating from the lung to the brain and crossing the blood-brain barrier (BBB) are among the main unsolved questions in the field (Del Poeta and Casadevall, 2012; Liu et al., 2012). As such, *C. neoformans* is an important model pathogen to unravel the fundamental question of how a microorganism is able to cross an apparently intact BBB. Many studies that use quantification of the fungal load or histological analysis of infected tissues have addressed the importance of specific virulence factors and immune cells in dissemination and BBB crossing (Charlier et al., 2009; He et al., 2012; Kaufman-Francis et al., 2018). In such experimental setups, the tissues of interest are isolated from euthanized animals at predefined time points. These post-mortem studies can be biased by inter-animal differences and do not permit longitudinal follow-up of disease dissemination in the context of living animals. While some attempts have been made to tackle this problem by using semi-invasive techniques (Shi et al., 2010; Tenor et al., 2015), sensitive, non-invasive approaches are needed to address this dynamic process in individual subjects throughout the entire disease period.

Over the last two decades, bioluminescence imaging (BLI) has emerged as a powerful tool to track cells of interest in animal models. The technique is based on the sensitive detection of photons that are generated by the oxidation of a substrate through a reaction catalyzed by a luciferase enzyme. Genetically engineering the pathogen of interest to express a luciferase gene allows for real-time visualization of the extent and location of infections in living animals (Avci et al., 2018). Owing to its non-invasive nature, BLI can monitor infection in individual animals during the entire course of the disease (Andreu et al., 2011). Thereby, it provides longitudinal information and better handles issues of inter-animal variations in spatial and temporal disease progression that are associated with traditional techniques for the quantification of the microbial load in isolated organs.

Since the first successful application of BLI in a *Salmonella* model, various bioluminescent bacteria, viruses and parasites have been generated (Avci et al., 2018; Contag et al., 1995; Hutchens and Luker, 2007). In comparison, BLI of fungal pathogens has to deal with some additional difficulties: potential barriers such as the fungal cell wall or cryptococcal capsule may hamper uptake of substrates, and the hypoxic environment in infectious niches may limit the amount of oxygen required to produce light (Avci et al., 2018; Brock, 2012; Doyle et al., 2006; Ibrahim-Granet et al., 2010; Vande Velde and Wiehr, 2017)*.* Nonetheless, BLI was successfully used to monitor superficial infections with the ascomycetes *Aspergillus* and *Candida* in models of subcutaneous, vaginal, oropharyngeal or biofilm-related catheter infection (Donat et al., 2012; Enjalbert et al., 2009; Mosci et al., 2013; Vande Velde et al., 2014). Challenges related to imaging of deeply located infections have been overcome, allowing detection and treatment monitoring of disseminated candidiasis and pulmonary aspergillosis (Brock et al., 2008; Galiger et al., 2013; Ibrahim-Granet et al., 2010; Jacobsen et al., 2014; Poelmans et al., 2018; Vande Velde et al., 2018). Besides codon optimization of reporter genes, the use of a red-shifted firefly luciferase can further increase the sensitivity for detecting deep-seated infections due to reduced light absorption by hemoglobin (Dorsaz et al., 2017). However, to date, *in vivo* BLI has not been applied to study infections caused by basidiomycete fungi such as *Cryptococcus* species*.*

While BLI offers excellent pathogen specificity and high sensitivity, other preclinical imaging techniques can provide additional information on how disease affects the organs. Both micro-computed tomography (µCT) and magnetic resonance imaging (MRI) can provide anatomical images with an excellent three-dimensional (3D) spatial resolution. Previous work has demonstrated the usefulness of CT and MRI to study lung disease progression in models of aspergillosis and cryptococcosis (Poelmans et al., 2016; Vande Velde et al., 2016). MRI has excellent soft-tissue contrast for brain imaging, and was successfully used in rats, showing hyperintense cryptococcal brain lesions on T2-weighted images (Himmelreich et al., 2002; Pai et al., 2009).

In this work, we have generated the first bioluminescent *C. neoformans* strain by integrating a codon-optimized, red-shifted, thermostable firefly luciferase gene in the previously described safe haven locus of the *C. neoformans* KN99α genome (Arras et al., 2015). We evaluated the use of BLI for the *in vivo* detection of *C. neoformans* infections in different murine models, including both systemic inoculation and the physiologically more relevant inhalation routes. By using a novel combination of BLI, lung μCT and brain MRI in models of cryptococcosis, we defined the variable time profile of the progression of dissemination from lung to brain in individual animals, leading to unprecedented observations about the timing of dissemination to the brain in relation to the development of lung disease.

## RESULTS

### *In vitro* characterization of the bioluminescent *C. neoformans* strains confirms the red-shifted emission of light

**Fig. 1.**
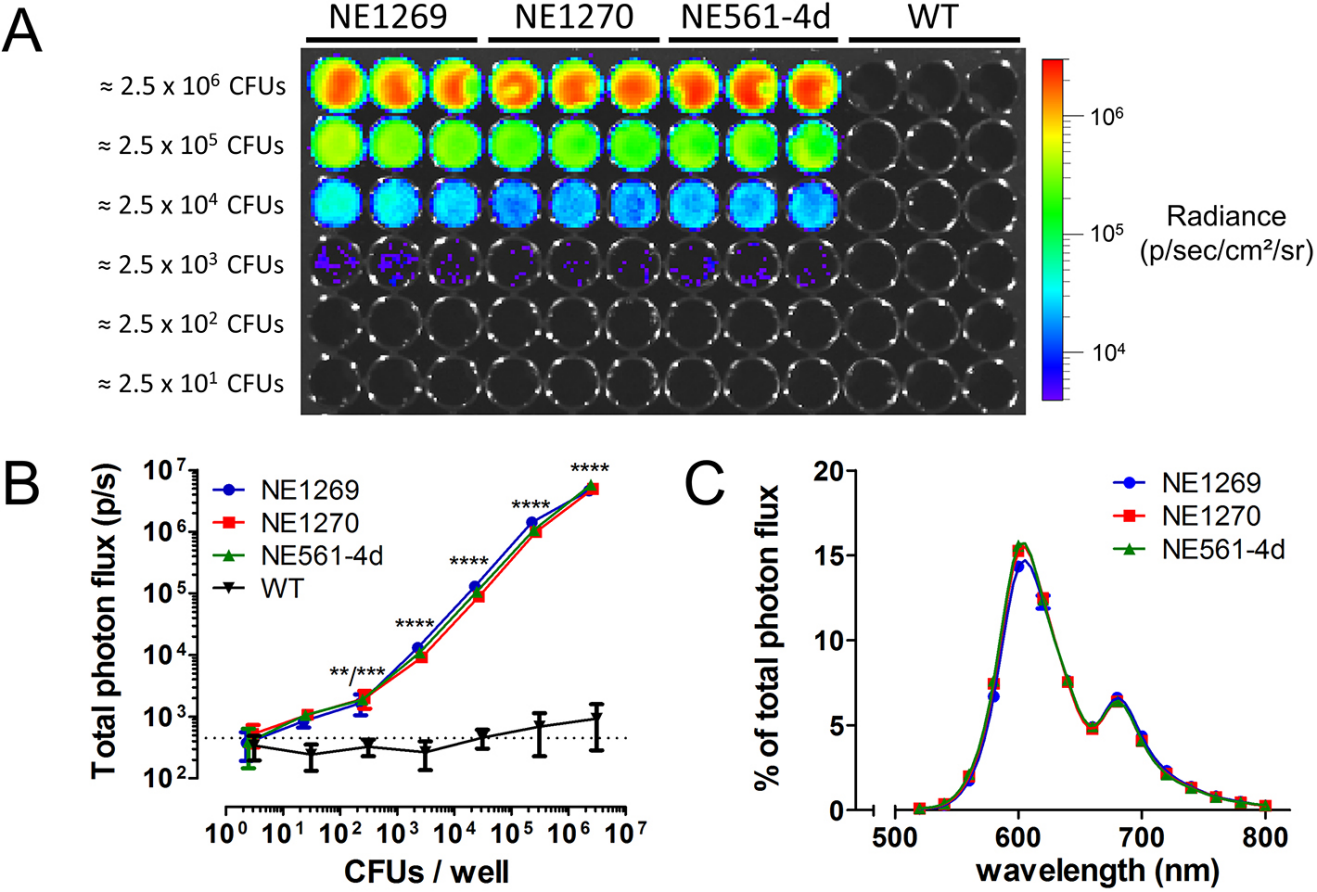
**Characterization of the bioluminescence emitted by the *C. neoformans* KN99α strains expressing the codon-optimized, red-shifted luciferase.** Serial 10-fold dilutions of the three selected transformants and the wild-type strain (WT) were imaged after addition of D-luciferin. (A) All three transformants showed comparable photon emission, whereas no signal was detected for the WT strain. (B) Quantification of bioluminescence showed that cells could robustly be detected starting from 250 CFUs per well. (C) Analysis of the emission spectra of the transformants confirmed a red shift of the emitted light. All data are represented as mean±s.d.; **P<0.01; ***P<0.001; ****P<0.0001; two-way ANOVA with Bonferroni post-test, compared to average signal from WT strain (dotted line). CFU, colony-forming unit.

We generated a bioluminescent *C. neoformans* KN99α strain by integrating a construct containing a codon-optimized, red-shifted and thermostable firefly luciferase (*CnFLuc*) gene in the previously described genomic safe haven site, in order to minimize potential alterations in the virulence profile of the original strain (Arras et al., 2015). An intron sequence was additionally placed in the synthetic gene sequence to avoid degradation of intron-less transcripts (Goebels et al., 2013). This artificially introduced intron sequence causes a frameshift if not correctly processed. Therefore, only a correct intron splicing results in the production of a functional luciferase. Indeed, three independent transformants showed highly comparable emission of bioluminescence (Fig. 1A) that was detectable starting from approximately 250 viable fungal cells *in vitro* (Fig. 1B). Spectral analysis confirmed the red-shifted emission of light, with a maximum around 600 nm and a second, smaller emission maximum around 680 nm (Fig. 1C). Introduction of the luciferase reporter construct did not alter the growth rate of the strains (Fig. S1). Strain NE1270 was selected for all subsequent *in vivo* experiments and is referred to as KN99α-CnFLuc. Infection with wild-type *C. neoformans* (H99) caused lung and brain disease in mice comparable to KN99α-CnFLuc with respect to weight loss, fungal load and presentation of the disease on brain MRI or lung μCT (Figs S2 and S3), indicating that the bioluminescent strain had similar *in vivo* virulence as the parental strain.

### BLI showed rapid dissemination to the brain and other organs in the intravenous model

**Fig. 2.**
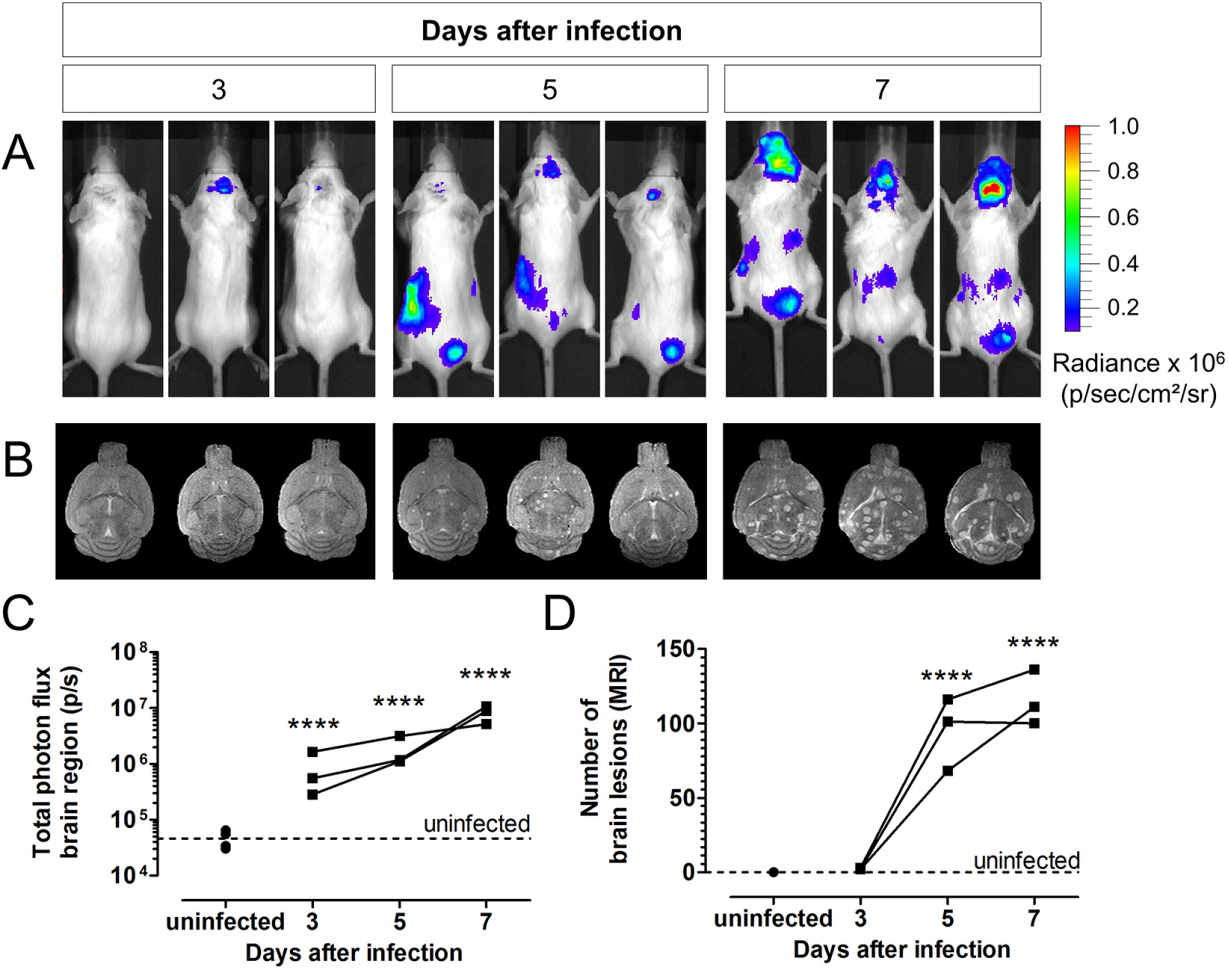
***In vivo* BLI and MRI of disseminated cryptococcosis.** (A) *In vivo* BLI of tail-vein-infected mice (*n*=3, 50,000 cells of KN99α-CnFLuc) on day 3, 5 and 7 post-infection (p.i.) showed rapid spreading of infection to the brain and abdominal region. (B) T2-weighted brain MR images of the same infected mice showed the presence of hyperintense brain lesions from day 5 onwards. (C) The total photon flux determined from the brains of these mice increased significantly starting from day 3 p.i. compared to uninfected controls (*n*=4). (D) The number of brain lesions detectable on the MR images increased starting from day 5 p.i. Dots and lines represent values for individual animals. ****P<0.0001, two-way RM ANOVA with Bonferroni post-test.

To evaluate the suitability of *in vivo* BLI to detect brain infection resulting from disseminated disease, mice were injected intravenously with 50,000 bioluminescent *C. neoformans* cells. Longitudinal BLI scans showed a rapid spreading of the fungus to the brain (Fig. 2A). MRI scans of the brain demonstrated the development of multiple hyperintense lesions in the brains of infected animals (Fig. 2B). Quantification of the BLI results indicated a significant increase in the bioluminescent signal from day 3 post-instillation (p.i.) (Fig. 2C), whereas brain MRI could only visualize lesions starting from day 5 p.i. (Fig. 2D). Furthermore, we observed signals from the abdominal region, including the kidney and bladder region. *Ex vivo* BLI and colony-forming unit (CFU) analysis of the isolated organs confirmed this observation by the presence of a high number of fungal cells in the kidneys and spleen (Fig. S4).

### *In vivo* BLI and lung µCT demonstrated differences in progression of lung disease upon intranasal inoculation with high or low infectious doses

**Fig. 3.**
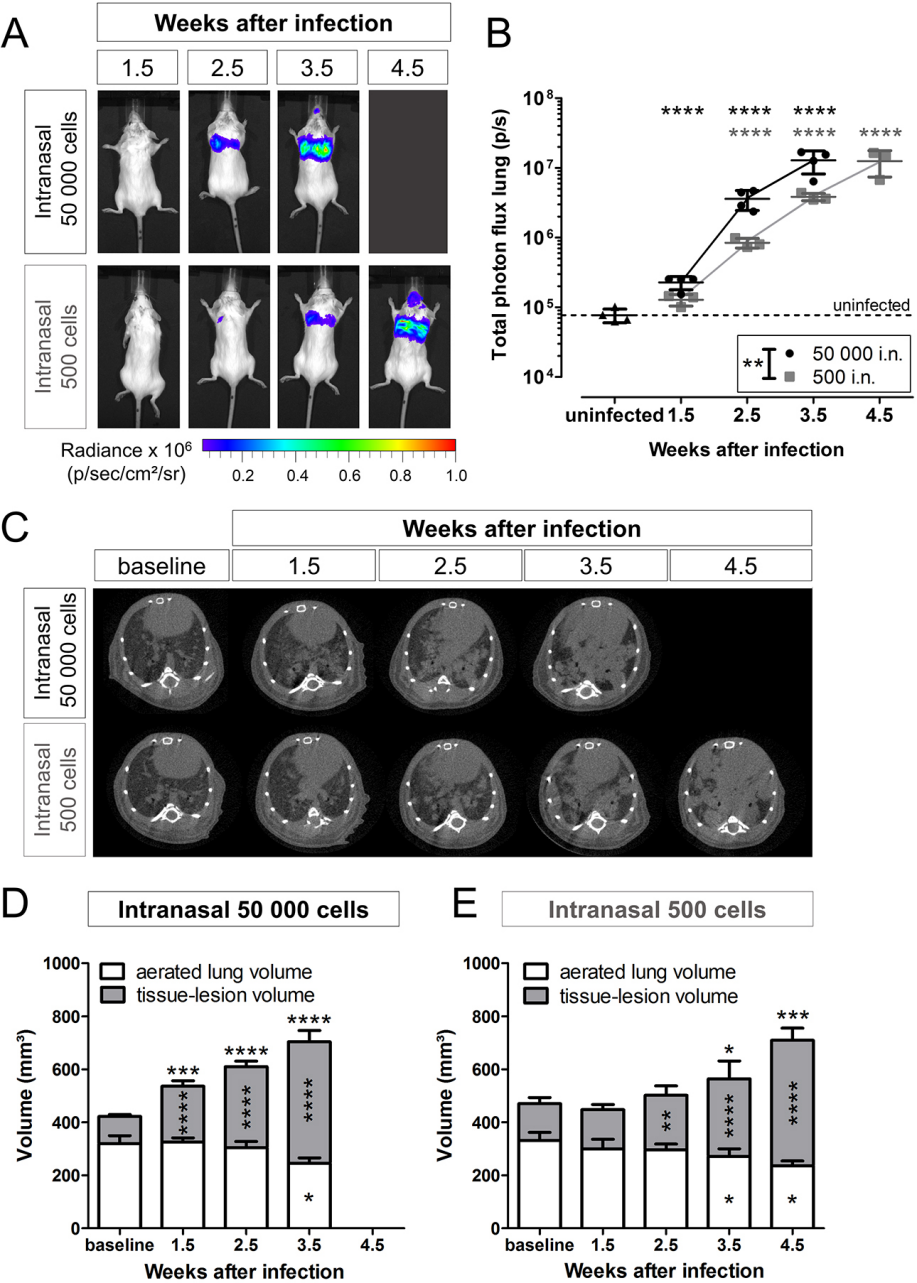
**BLI and μCT of pulmonary infection upon intranasal inoculation.** (A) *In vivo* BLI showed the development of a progressive pulmonary and, to a minor extent, nasal or sinus infection upon intranasal (i.n.) inoculation with 50,000 (*n*=4) or 500 (*n*=3) *C. neoformans* KN99α-CnFLuc cells. (B) Quantification of the bioluminescence signal from the lung region demonstrated significantly different progression of infection in both inoculum size groups (group effect *P*=0.0033). (C) Transversal lung μCT images showed the deposition of dense lesion tissue (gray) in the normally aerated (black) regions of the lungs. (D,E) During infection, the tissue-lesion volume (quantified from CT images) increased in both the high (D) and low inoculum (E) group (both *n*=4) and was significantly different between both groups (*P*=0.0227). Additionally, the aerated lung volumes decreased (group effect not significant). The total lung volume, composed of both the tissue-lesion and aerated lung volume, increased during the later time points (group effect *P*=0.0121). Graphs show individual data points and/or mean with s.d. for every time point. *P<0.05; **P<0.01; ***P<0.001; ****P<0.0001, two-way RM ANOVA with Bonferroni post-test, comparison with uninfected controls (BLI, *n*=4) or baseline (CT). Significance for total lung volume is indicated on top of the bars.

To evaluate the suitability and sensitivity of BLI to monitor pulmonary infections, mice were infected via the intranasal route with 50,000 (high inoculum) or 500 (low inoculum) *C. neoformans* KN99α-CnFLuc cells. Both groups developed a progressive pulmonary infection that was visualized by BLI (Fig. 3A). Infection progressed significantly more rapidly in animals infected with the high inoculum (Fig. 3B).

In parallel, we cross-validated the BLI results in the lung with µCT. Animals developed multiple lung lesions that appeared as gray patches against the dark background of the aerated part of the lung (Fig. 3C). The quantified tissue-lesion volume, which includes both normal lung tissue and the additional lesion tissue due to infection, increased gradually after instillation with 50,000 (Fig. 3D) or 500 (Fig. 3E) fungal cells. During infection, and especially in the later stages of disease, the aerated lung volume decreased, while the total volume of the lungs increased (Fig. 3D-E).

Furthermore, we assessed the potential of these imaging techniques to differentiate between the high- and low-inoculum groups. Both BLI and µCT were able to demonstrate a significant difference in the progression between both groups (Fig. 3). When compared with additional cross-sectional data at week 3.5 p.i., the bioluminescence signal and tissue-lesion volume on µCT was significantly different between both inoculum groups (Fig. S5). In contrast, CFU counts could not discriminate between the fungal loads in both inoculum groups. Overall, BLI and µCT data indicate that the development of detectable disease in the low-inoculum group is delayed by approximately 1 week compared to the high-inoculum group, while having a similar progression rate.

### Sensitive detection of dissemination to the brain in the intranasal model was hampered by strong lung and nose signals

**Fig. 4.**
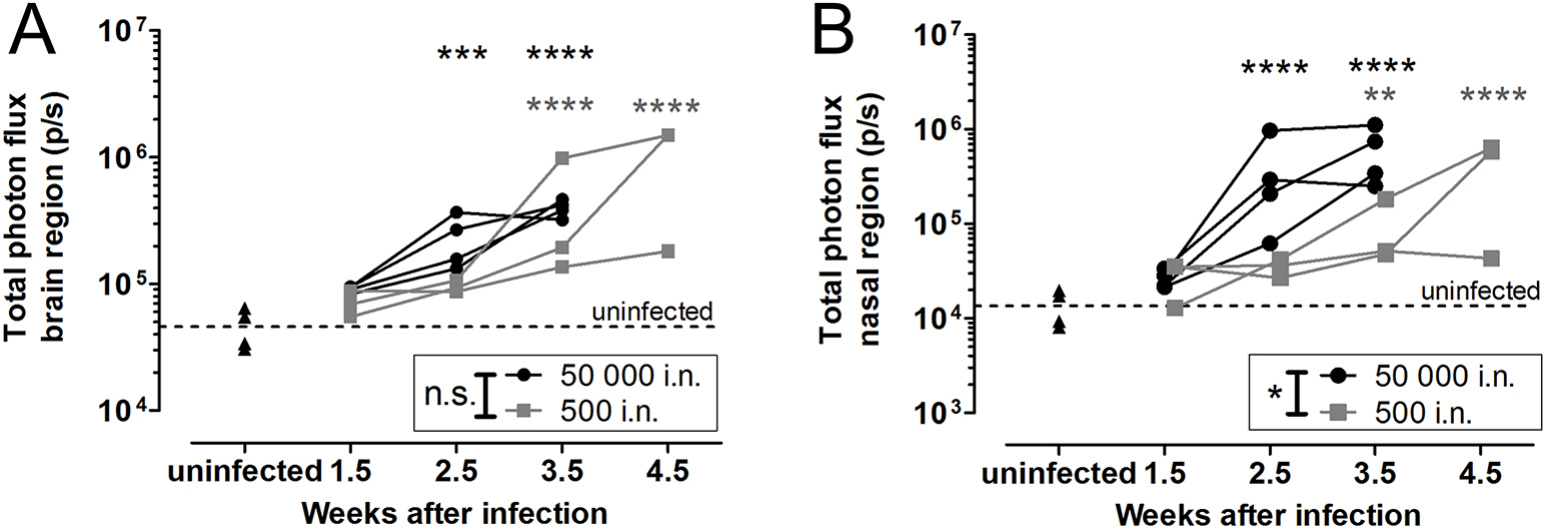
**BLI of the brain region and nasal region after intranasal inoculation.** (A) Bioluminescence signal intensity from the brain region increased as infection progressed, with no significant differences between both inoculum groups [50,000 (*n*=4) or 500 (*n*=3) KN99α-CnFLuc cells, *P*=0.0742]. (B) A similar trend was observed for the nasal region, with a significantly more pronounced nasal signal in the higher-inoculum group (*P*=0.0131). **P<0.01; ***P<0.001; ****P<0.0001; two-way RM ANOVA with Bonferroni post-test in comparison to uninfected controls (*n*=4). i.n., intranasal.

Next, we investigated whether we could detect evidence of brain involvement in the intranasal inoculation model in order to establish a timeframe for the progression from lung to brain disease. During the course of infection, the bioluminescence signal originating from the brain area increased steadily and was significantly higher than the background signal starting from week 2.5 p.i. in the high-inoculum or week 3.5 p.i. in the low-inoculum group (Fig. 4A). In parallel, the signal from the nasal area increased, indicating colonization and multiplication of the yeast in the nasal region of animals (Fig. 4B).

Since BLI is associated with a limited spatial resolution, we subsequently studied the origin of the bioluminescence signal in more detail using a combination of *in vivo* imaging and *ex vivo* techniques (Fig. S6). CFU analysis demonstrated the presence of cryptococci in the brain 3.5 (high inoculum) or 4.5 (low inoculum) weeks after infection. Since neither MRI, *ex vivo* BLI nor CFU analysis could confirm brain infection in animals prior to week 4.5 for the low-inoculum group, the increased *in vivo* BLI signal from the brain region at week 3.5 likely resulted from light emission or scattering from neighboring infected tissues. Although *in vivo* BLI enabled reliable detection of advanced brain infection, the quantification of the weak bioluminescence in brains with low fungal load was confounded by the strong signal from the lung and nose infection in the intranasal infection model.

### Using the endotracheal model, BLI provided robust quantification of the dissemination to the brain for individual animals

**Fig. 5.**
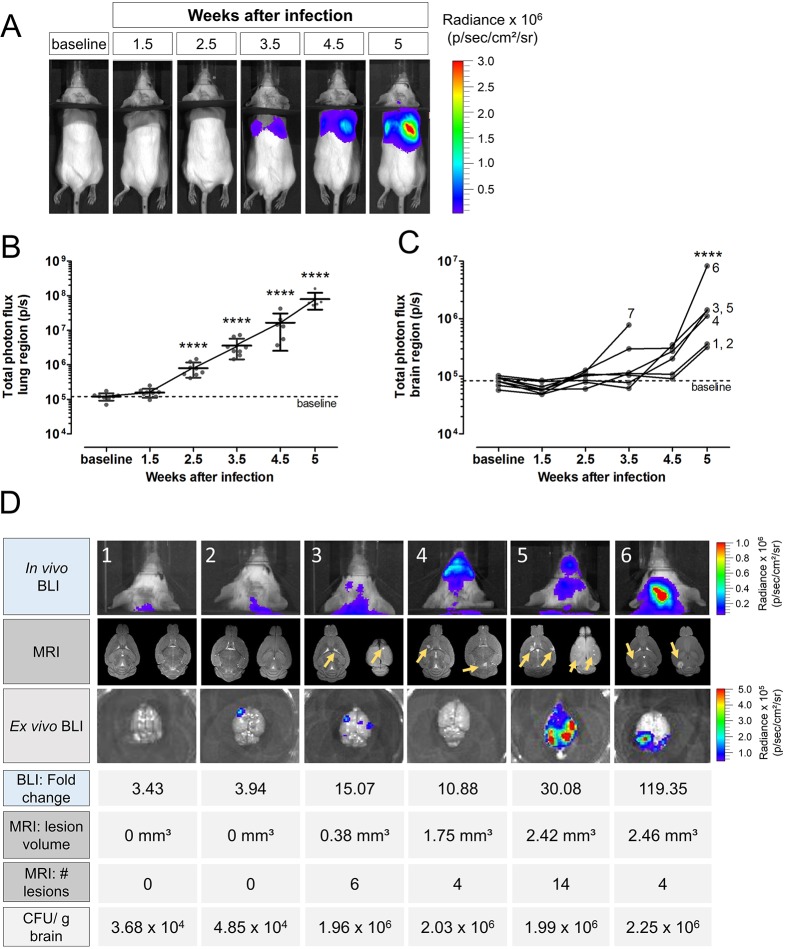
**Lung and brain infection in the endotracheal model as visualized by BLI and MRI.** Mice (*n*=12) infected with 500 *C. neoformans* KN99α-CnFLuc cells via the endotracheal route were scanned longitudinally using *in vivo* BLI and MRI. Three animals did not develop infection and, for one other animal, BLI-visible infection started only 1 month after inoculation (data not shown and excluded from analysis). Two animals required sacrificing after 3.5 weeks. (A) Representative BLI pictures of an individual mouse developing a lung infection in the absence of a nasal signal as observed in most animals. A black partition was placed over the animal's neck to separate light from the lung and brain regions. (B) Time-dependent increase of total photon flux from the lung region demonstrated the progression of the pulmonary infection. (C) The BLI signal intensity from the brain region remained at baseline values during early infection, but significantly increased at week 4.5 and 5, indicating dissemination to the brain. For animal 7, bioluminescent signal was observed without detectable CFUs in the brain, which can be explained by nasal colonization without brain involvement. Graphs show dots per individual animal with mean and s.d. or individual lines per animal. The numbers indicate the corresponding animal number. ****P<0.0001, RM one-way ANOVA with Bonferroni post-test compared to baseline. (D) *In vivo* BLI images, MR images of the brain (two slices of the 2D scan per animal) and *ex vivo* BLI images with the corresponding values of fold change in BLI signal (compared to baseline), MRI lesion number and volume, and CFUs/g brain at week 5 showed the extent of infection for individual animals. Animals 1 and 2 (10^4^ CFUs/g brain) did not present with a visual signal on BLI or MRI but had a 3- to 4-fold increase in the *in vivo* BLI signal at week 5. Animals 3, 4 and 5 had multiple small lesions on MRI (yellow arrows) and 10- to 30- fold increased BLI signal. Animal 6 presented with one localized hotspot on BLI corresponding to a large lesion on MRI.

To avoid potentially confounding signals from the nasal region, our next step was to use a model of endotracheal instillation. In addition, we placed a black partition over the neck of the animal to separate the bioluminescence of the lung region from the bioluminescence of the brain region (Fig. S7). As in the intranasal model, BLI showed that animals infected with 500 (Fig. 5A,B) or 50,000 (Fig. S8) cells developed a progressive lung infection. Lung µCT demonstrated a comparable evolution in the development of lung lesions (Fig. S9). Quantification of the bioluminescence signal from the brain area in the low-inoculum group showed values close to baseline during the early stages of infection (Fig. 5C). At 4.5 and 5 weeks p.i., we observed a marked increase in the brain bioluminescence signal intensities for all remaining animals (Fig. 5C). Some animals showed colonization of the nasal area, but in most animals, the bioluminescence in this area remained close to baseline (Fig. S10).

We subsequently evaluated the *in vivo* BLI, brain MRI and *ex vivo* BLI results of individual animals (Fig. 5D). Animals with a low fungal load in the brain (order of 10^4^ CFUs/g brain, animals 1 and 2, Fig. 5D) showed an approximate 3.5-fold increase in the *in vivo* BLI signal at week 5 compared to baseline, but no lesions could yet be detected on MRI. Animals with a fungal load in the order of 10^6^ CFUs/g brain (animals 3-5, Fig. 5D) showed multiple lesions on MRI and a 10- to 30-fold increase in BLI signal. Animal 6 presented with a large localized lesion on MRI and a hotspot of light emission on the BLI images (Fig. 5D). Overall, we observed that animals with earlier dissemination to the brain corresponded to those animals that had more extensively developed pulmonary infection.

### The findings from BLI or μCT/MRI are representative of the fungal load in the brain and lungs

**Fig. 6.**
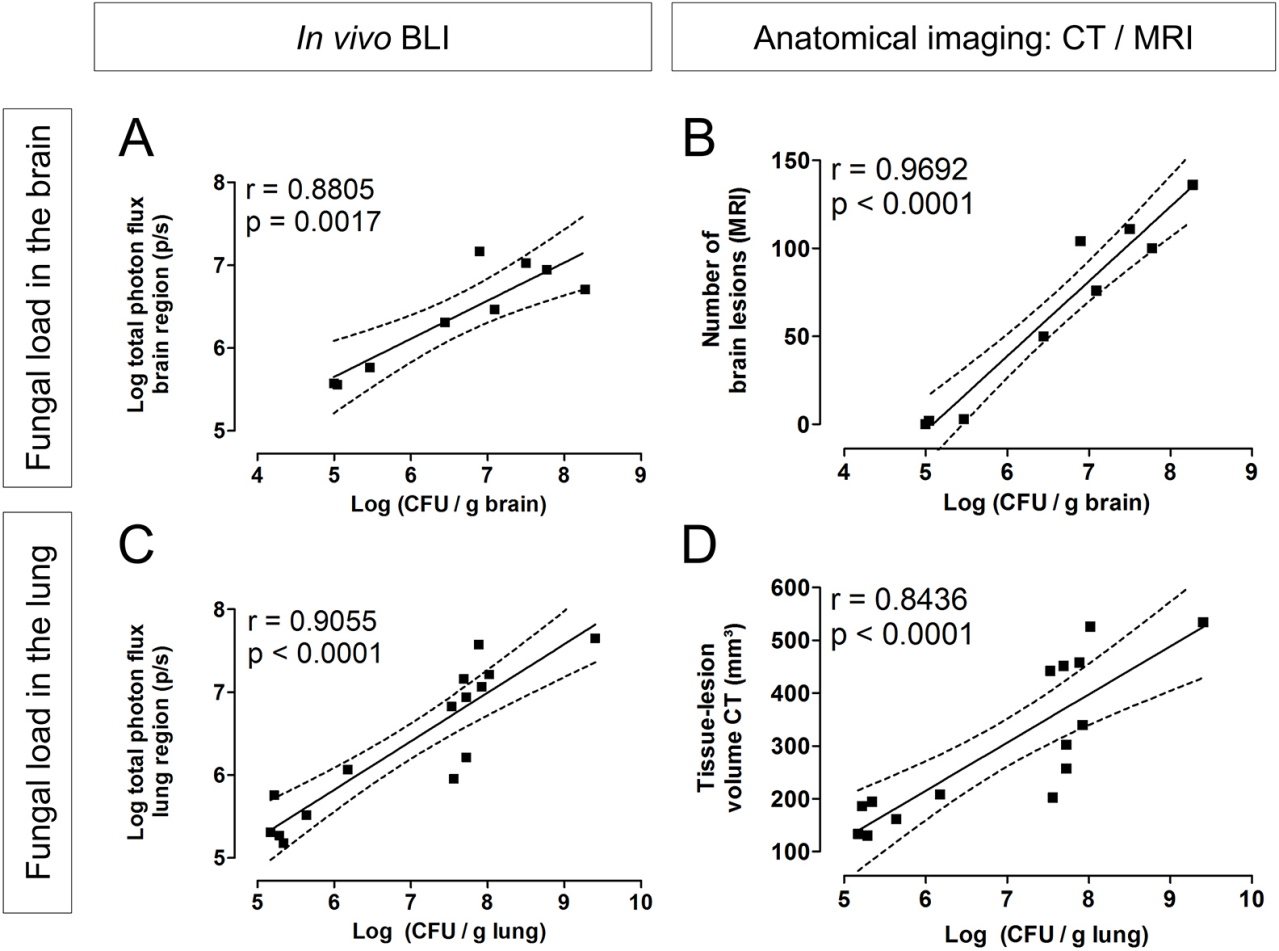
**Correlation of imaging readouts with fungal load in lung and brain.** (A,B) Animals infected intravenously (50,000 *C. neoformans* KN99α-CnFLuc cells) were scanned using brain MRI and BLI, and were subsequently sacrificed for CFU counting of the brain at day 3, 5 or 7 (*n*=3 each). Both the *in vivo* BLI signal (A) and the number of brain lesions on MRI (B) showed a strong correlation with the fungal load in the brain. (C,D) Mice infected with 500 *C. neoformans* KN99α-CnFLuc cells via the intranasal route were scanned (BLI and lung μCT) and subsequently sacrificed for CFU counting of the lung at 1.5, 2.5, 3.5 (*n*=3 per time point) or 4.5 (*n*=6) weeks p.i. *In vivo* bioluminescence (C) and the tissue-lesion volume quantified from μCT (D) correlated well with CFUs from lung tissue. Dots represent individual animals. The dashed lines indicate the 95% confidence bands of the regression curve with the corresponding Pearson correlation coefficient. Datasets include end-point data from the longitudinal studies (day 7 for intravenous or week 4.5 for intranasal) and additional cross-sectional data from intermediate time points.

Finally, we aimed to evaluate whether our imaging findings are truly representative for the fungal load in the organs of interest. Using data from the cross-sectional and longitudinal studies described above, we correlated the readouts of a specific time point with the corresponding CFU values in the brain (intravenous model) or lung (intranasal model). Both BLI (total photon flux in the brain region; Fig. 6A) and MRI (number of lesions in the brain; Fig. 6B) results correlated well with the CFUs in the brain. Similarly, a good correlation of CFUs with BLI (total photon flux of the lungs; Fig. 6C) and μCT (tissue-lesion volume in the lung; Fig. 6D) results could be established. Most animals had 10^7^-10^8^ CFUs in the lungs when having advanced pulmonary infection, and CFU counting was limited in discriminating in the extent of infection between these animals. In contrast, μCT and BLI could assess the inter-animal differences and showed a good correlation in this range (Fig. S11). In addition, we found good correspondence between the BLI results and the readouts obtained from MRI (Fig. S11).

## DISCUSSION

In this study, we have established a temporal profile for brain dissemination upon systemic and pulmonary fungal infection with the model pathogen *C. neoformans*. To that end, we have created the first bioluminescent *C. neoformans* strain, expressing a red-shifted, thermostable version of the firefly luciferase. The introduced intron sequence was correctly spliced, as all transformants produced bioluminescent signals after the addition of the substrate D-luciferin. Moreover, the reporter strains were successfully applied for BLI of infection in murine models of cryptococcosis. This result indicates that BLI is not only applicable to ascomycetes such as *Aspergillus* and *Candida* species, but also to basidiomycetes. Using BLI, we were able to monitor the spatial and temporal distribution of infection following intravenous, intranasal or endotracheal instillation. In combination with μCT of the lung and MRI of the brain, this allowed us to define a timeframe for the dissemination of cryptococcosis from the lung to the brain for each individual animal.

The major aim of our study was to gain a better understanding of the dynamics of the dissemination of cryptococcosis to the brain after a pulmonary infection. The brain is a challenging organ for BLI because substrate bioavailability is relatively limited and the amount of detectable light is reduced by the overlying skin and skull (Aswendt et al., 2013; Berger et al., 2008). To optimize the *in vivo* detection and increase sensitivity, we generated a modified luciferase that emits light at longer wavelengths (600 and 680 nm) than the natural firefly luciferase (560 nm), leading to a deeper light penetration through the tissue and a reduced absorption by hemoglobin. This permitted reliable tracking of a minimum of 10^4^ cells under *in vivo* conditions in different organs, allowing the non-invasive visualization of the early onset of disease development in individual animals.

By using a combination of BLI and MRI, we could define the time and extent of brain infection and damage with high spatial and temporal resolution. Upon intravenous injection of cryptococci, fungi were detected in the brain within a short timeframe of a few days. Initial detection of brain infection using BLI in the intranasal model was influenced by the strong signals from the nasal region, but reliable detection of brain infection was possible for animals infected via the endotracheal route. Spreading to the brain in the physiologically more relevant inhalation models was variable between animals in terms of location and timing. In the low-inoculum intranasal model, no fungal cells were recovered from the brain at 3.5 weeks p.i. or prior to that. The presence of brain BLI signal in the endotracheal model was detected during weeks 4-5, with a rapid increase in the signal at the last time point prior to sacrifice. These findings indicate that dissemination to the brain is probably a late-stage event that occurs somewhere between 3.5 and 4.5 weeks after the instillation with 500 fungal cells.

We observed that animals with a higher fungal load in the lungs developed brain infection more rapidly, suggesting that the likelihood for dissemination to the brain is influenced by the fungal burden in the lungs rather than the duration of fungal presence. Nonetheless, the development of extensive pulmonary disease in the high-inoculum group limited the time for observation of brain involvement, as these animals frequently succumbed due to pulmonary disease before the development of substantial brain infection.

In comparison to intravenous bolus injection of a relatively large inoculum of cryptococci, inhalation models are more representative of the natural route of infection and permit the study of factors influencing the initial exit from the lungs as well as BBB crossing. However, the substantial inter-animal variability in these models makes the onset and extent of brain infection unpredictable and therefore difficult to handle in cross-sectional studies that use only end-point CFU analysis or histology. Since BLI can be longitudinally performed with a time resolution of 1 day, the day of dissemination can now be defined for individual animals, enabling the study of BBB crossing in the more physiological context of prior lung infection. Its implementation in *Cryptococcus* research will result in more appropriate selection of relevant time points to apply complementary techniques at the cellular level, such as intravital microscopy or histological analysis, leading to novel insights into the pathogenesis of cryptococcosis (Kaufman-Francis et al., 2018; Shi et al., 2010; Vanherp et al., 2018). In addition, time point selection for the assessment of novel therapeutic approaches can be based on physiologically relevant time points rather than on an arbitrary basis.

Although BLI is a sensitive technique that is highly specific for the detection of viable pathogens of interest, defining the exact location of the infection can be more challenging due to the limited spatial resolution. Furthermore, BLI can over- or underestimate the extent of infection because the surface-weighted signal is dependent on the depth of the bioluminescent source within the subject. In addition, the availability of the cofactor oxygen and the substrate D-luciferin is potentially limited in larger lesions and may depend on disease-related changes in BBB integrity (Ayzenberg et al., 2015; Brock et al., 2008). In our study, we overcame these inherent limitations of BLI by combining the technique with anatomical imaging of the lung and brain. Although BLI proved to be more sensitive in detecting the early brain infection, MRI allowed the identification and 3D localization of cryptococcomas. By combining the high specificity and sensitivity of BLI with the resolution and 3D anatomical information of MRI, we obtained a more accurate assessment of the extent of brain infection and damage for each individual animal. Nonetheless, even without the addition of technically more demanding and less readily available anatomical imaging, BLI correlated well with CFU analyses and successfully enabled quantification of disease progression and dissemination with sufficient temporal and spatial resolution, despite possible limitations in substrate availability.

In the lungs, BLI allowed rapid and sensitive assessment of infection. Its combination with μCT revealed changes in aerated and total lung volumes that potentially reflect the underlying physiological mechanisms (Vande Velde et al., 2016). Animals in which initiation of infection had failed were easily identified and excluded from the study, leading to reduced inter-animal variability. Using only a small number of animals, both techniques could discriminate between the progression of pulmonary infection in the high- and low-inoculum groups, thanks to the increased statistical power of repeated-measures designs.

BLI detected the spatial distribution of infection following different routes of inoculation and thereby also revealed target sites outside of the lung or brain. Firstly, signals from the abdominal region in the intravenous model originated from the spleen and kidneys. The presence of fungal cells in these organs has also been demonstrated in other studies and indicates that intravenous injections induce a widespread systemic infection (Capilla et al., 2006; Ngamskulrungroj et al., 2012). Secondly, we observed strong signals from the nasal region after intranasal instillation. Colonization of the nasal mucosa and sinuses associated with this administration route has previously been shown in murine models using other bioluminescent microorganisms (Ibrahim-Granet et al., 2010; Warawa et al., 2011). One study has also reported on the persistence of *Cryptococcus* in the nasal passages of infected mice (Anderson and Sagha, 1988). Endotracheal instillation resulted in the absence of signals originating from the nasal region for most but not all animals, confirming that this route of inoculation limits but does not completely exclude colonization of the nasal and sinus region (Santry et al., 2017). Nonetheless, intratracheal instillation of *Cryptococcus* (Muhammed et al., 2012), or non-surgical alternatives such as intrapharyngeal instillation (Ngamskulrungroj et al., 2012) or the endotracheal instillation introduced here, are valuable alternatives to bypass the nose in case this interferes with readouts.

In infectious diseases research, evaluating and quantifying the number of microorganisms in specific organs is of major importance. The generally applied technique of CFU counting, based on serial dilutions of tissue homogenates, can only be done as end-point measurement and is prone to variability. Our *in vivo* imaging readouts provided an excellent correlation with the fungal load in the lung and the brain. BLI and μCT allowed for a better assessment of the extent of highly progressed infection in the lung than CFU counting, indicating that the dynamic range of BLI and μCT is potentially more suitable for evaluating advanced infections. Furthermore, CFU analysis was unable to discriminate between the lung fungal load in the high- and low-fungal-inoculum groups, while both BLI and CT yielded significant differences between these groups.

These results show that the readouts that we obtain via non-invasive preclinical imaging can provide highly representative, quantitative and truly longitudinal information about the fungal burden in organs of interest and, most importantly, in live animals. Furthermore, the use of non-invasive techniques in longitudinal studies brings important advantages: a reduced number of animals needed in research; increased statistical power; and accurate assessment of the variability in the infection status of each subject. As previously shown for other fungi, BLI can also readily be applied for evaluating the *in vitro* or *in vivo* efficacy of antifungal drugs (Binder et al., 2018; Galiger et al., 2013; Jacobsen et al., 2014; Poelmans et al., 2018; Vande Velde et al., 2018). However, careful interpretation of treatment results is warranted as the limited presence of oxygen, ATP or luciferin in necrotizing tissue can also influence the light-generating reaction. This non-invasive imaging approach can therefore be an important tool to evaluate dynamic processes such as disease progression, dissemination and therapy response in living animals throughout the whole course of the disease.

In conclusion, we have created the first bioluminescent *C. neoformans* strain and successfully monitored the spatial and temporal distribution of infection in living animals using BLI. As a stand-alone technique or further complemented by anatomical imaging of the lung and brain, BLI allowed us to narrow down the timeframe of the progression from pulmonary to cerebral cryptococcosis for individual animals using physiologically relevant inhalation models. Applying this technique in transgenic or immunocompromised animals, or creating bioluminescent *Cryptococcus* mutants, will aid the identification of pathogen or host factors that enable *C. neoformans* to exit the lungs and disseminate to the brain. This will not only advance our understanding of the pathogenesis of the disease, but can also assist in the identification and validation of novel potential therapeutic targets.

## MATERIALS AND METHODS

### Generation of a synthetic codon-optimized, red-shifted, thermostable firefly luciferase for expression in *C. neoformans*

The protein sequence of the native firefly luciferase was used as a template to introduce amino acid exchanges at positions T214A, A215L, I232A, S284T, F295L and E354K for generating a red-shifted thermostable luciferase with enhanced properties for *in vivo* imaging (Dorsaz et al., 2017). To deduce a codon-optimized luciferase gene sequence, a set of 40 highly expressed genes from *C. neoformans* H99 was selected as template for a codon usage table and its accompanied di-codon adaptation index (dCAI) by using the dCAIoptimizer (Jacobsen et al., 2014). The mutated firefly luciferase protein sequence was then back-translated into a codon-optimized DNA sequence. To further prevent problems of reduced mRNA accumulation deriving from intron-less gene sequences in *C. neoformans* (Goebels et al., 2013), a 55 base pair (bp) intron sequence deriving from the *C. neoformans* glyceraldehyde-3-phosphate dehydrogenase gene was introduced at position 70 downstream to the ATG start codon. As this intron causes a frameshift and early termination of translation if not spliced from the transcript, only correctly processed mRNA results in the production of a functional luciferase. To regulate gene expression, the synthetic luciferase gene (here referred to as *CnFLuc*; GenBank accession number MH920367) was fused by *in vitro* recombination (InFusion HD cloning kit, Takara/Clontech) with a 1167 bp *EF1a* promoter and a 402 bp *TRP1* terminator sequence from *C. neoformans*.

### Generation of a bioluminescent *C. neoformans* strain

The plasmid pNE560 was constructed by cloning the 3277 bp synthetic construct containing the optimized luciferase-encoding gene in the ‘safe haven’ plasmid pSDMA25 (Arras et al., 2015) at the *Hin*dIII and *Cla*I restriction sites. After linearization using the *Pac*I restriction enzyme, the PNE560 plasmid was integrated in the *C. neoformans* KN99α genome by biolistic transformation (Toffaletti et al., 1993). The transformants were selected on nourseothricin-containing medium (200 µg/ml). Correct integration at the safe haven site was confirmed by multiplexed PCR using the primers UQ1768, UQ2962, UQ2963 and UQ3348, as previously described (Arras et al., 2015). Three independent transformants were selected (NE1269, NE1270 and NE561-4d) and stored at −80°C.

### Fungal culture

The wild-type H99, KN99α or bioluminescent *C. neoformans* strains were first grown on Sabouraud agar and then transferred to liquid Sabouraud medium (both Bio-Rad, Temse, Belgium). In both steps, incubation was for 2 days at 30°C. Fungal cells were harvested by centrifugation, followed by two washing steps with Dulbecco's phosphate-buffered saline (PBS; Gibco, Paisley, UK). Finally, the number of cells was counted using a Neubauer counting chamber (Mariënfeld superior, Lauda-Köningshofen, Germany) and diluted to the desired concentration in PBS. To confirm the number of CFUs, dilutions of the inoculum were plated on Sabouraud agar and incubated for 2 days at 30°C or 3 days at room temperature.

For the measurement of growth curves, the wild-type KN99α and transformed *C. neoformans* strains were inoculated in liquid Sabouraud medium. The optical density at 595 nm was measured for 48 h using a Wallac 1420 plate reader (Perkin Elmer, Turku, Finland). Four samples per strain were inoculated and measured in triplicate. The median value of the sterile medium was subtracted. Growth rates were analyzed by fitting a logistic growth model for every sample up to the measurement at 40 h.

### *In vitro* bioluminescence assay

Triplicate 10-fold serial dilutions of the harvested fungal suspension were prepared in a black 96-well plate (Nunc^®^ Microwell, Thermo Fisher Scientific, Merelbeke, Belgium). An equal volume of D-luciferin solution (final concentration 0.15 µg/ml in PBS; Luciferin-EF, Promega, USA) was added. Images were acquired using an IVIS Spectrum system (Perkin Elmer, Waltham, USA) with the Living Image software (version 4.5.2) and an exposure time of 1 min, F/stop of 1 and medium binning. Individual regions of interest (ROIs) were placed over each well for quantification of the total photon flux. Afterwards, the number of CFUs per well was determined by triplicate plating of serial dilutions on Sabouraud agar, cultured as specified above.

For analysis of the emission spectra from bioluminescent *C. neoformans* strains, bioluminescence of the fungal suspensions was measured using emission filters from 520 to 800 nm (step size 20 nm) and an exposure time of 30 s. The observed total photon flux per emission filter was expressed relative to the total photon flux in the filter ‘open’ setting and a cubic spline curve was fitted.

### Mouse models

All animal experiments were conducted in accordance with European Directive 2010/63/EU and approved by the Animal Ethics Committee of KU Leuven (P006/2017, P103/2012). Animals were housed in individually ventilated cages and received water and standard food *ad libitum*. Female BALB/c mice (internal stock KU Leuven or Envigo, Horst, The Netherlands) were infected at an age of 9-10 weeks. For model induction and imaging, mice were anesthetized using 1.5-2% isoflurane (Abbott Laboratories, Queensborough, UK) in 100% oxygen unless otherwise specified. Mice were sacrificed at predefined time points (cross-sectional studies) or when humane endpoints were reached (longitudinal studies).

#### Intravenous injection model

Animals (*n*=3) were infected by injection of 50,000 fungal cells in 100 µl of PBS via the tail vein (modified from Ngamskulrungroj et al., 2012). Bioluminescence and MR images were acquired on day 3, 5 and 7 after infection, after which animals were sacrificed for *ex vivo* BLI and CFU counting. Two additional animal groups (*n*=3 each) were only scanned on day 3 or 5 and sacrificed afterwards to obtain *ex vivo* BLI data and CFU counts for intermediate time points (cross-sectional study). Animals (*n*=3) infected with wild-type H99 were only scanned with MRI on day 3, 5 and 7 after infection, after which CFU analysis was performed for one animal.

#### Intranasal instillation model

Animals were instilled intranasally with 500 (*n*=4) or 50,000 (*n*=4) fungal cells, suspended in 20 µl PBS and divided over both nostrils (Vanherp et al., 2018). Animals were scanned weekly using BLI, MRI and μCT starting from 1.5 weeks after instillation (longitudinal study). Scans with the different modalities were spread over two consecutive days, e.g. 1.5 weeks indicated scans around day 10 or 11. One animal infected with 500 cells was excluded from the BLI analysis due to inefficient intravenous injection of luciferin. For cross-sectional studies, additional animals were infected with 500 cells (*n*=3 per group), scanned once by *in vivo* BLI, μCT and MRI after 1.5, 2.5, 3.5 or 4.5 weeks, and subsequently sacrificed for *ex vivo* BLI and CFU counting. For animals infected with wild-type H99 (*n*=5), only lung μCT and brain MRI was performed.

#### Endotracheal instillation model

A published oropharyngeal instillation protocol (Lakatos et al., 2006) was modified to avoid potential gastrointestinal exposure. First, the animal was injected intraperitoneally (i.p.) with a mixture of ketamine (45-60 mg/kg, Nimatek^®^, Eurovet Animal Health, Bladel, The Netherlands) and medetomidine (0.6-0.8 mg/kg, Domitor^®^, Orion Pharma, Espoo, Finland) to induce anesthesia. Subsequently, the animal was suspended by its upper incisors on an angled platform. A suspension of 500 or 50,000 fungal cells in 40 µl PBS was instilled endotracheally by placing a pipette tip near the tracheal entrance. Anesthesia was reversed by i.p. injection of atipamezole hydrochloride (0.5 mg/kg, Antisedan^®^, Orion Pharma). Animals infected with 500 cells (*n*=12) were scanned by using BLI and μCT at baseline and 1.5, 2.5, 3.5, 4.5 and 5 weeks after infection, with weekly brain MRI scans starting from week 2.5. Animals infected with 50,000 yeast cells (*n*=4) were scanned using lung μCT at baseline and after 1.5 weeks, and BLI at baseline and afterwards bi-weekly. The baseline BLI measurements from these four animals also served as uninfected controls in the intravenous and intranasal model.

### *In vivo* imaging

#### Bioluminescence imaging

Fur on the top of the head was clipped to optimize the detection of bioluminescence originating from the brain. A D-luciferin solution (15 mg/ml in PBS) was injected intravenously via the tail vein of the mouse at a dose of 126 mg/kg body weight. This injection route was chosen to improve the imaging sensitivity for the brain (Aswendt et al., 2013; Berger et al., 2008). Subsequently, animals were placed in a prone position in the IVIS Spectrum. Images were acquired continuously for a period of 20 min using an exposure time of 1 min per image, F/stop of 1, a subject height of 1.5 cm, field-of-view of 13.4 cm and medium binning. In the endotracheal model, a black partition was placed over the animal's neck (Fig. S7).

#### Micro-computed tomography of the lung

Mice were placed in a supine position in a small-animal µCT scanner (SkyScan 1278, Bruker micro-CT, Kontich, Belgium). Scans were acquired using the following parameters: isotropic voxel size of 50 µm, 1 mm aluminum filter, X-ray source of 50 kV, source current of 920 µA, an exposure time of 55 ms and a 0.9° rotation step with nine projections per position for a total angle of 180°, with a total acquisition time of approximately 10 min. During scanning, the respiratory rate of the animal was monitored using a video system.

#### Magnetic resonance imaging of the brain

MR images were acquired on either a 7 T preclinical MRI scanner with a 30 cm bore and a quadrature resonator (86 mm diameter) or a 9.4 T scanner with a 20 cm bore and a linearly polarized resonator (72 mm diameter), both in combination with an actively decoupled mouse brain surface coil (all Bruker Biospin, Ettlingen, Germany). Body temperature and breathing rate of the animals were monitored using a physiological monitoring system (Small Animal Instruments Inc., Stony Brook, NY, US). After obtaining initial localizer images, a 2D T2-weighted brain scan (RARE sequence) was acquired with the following parameters: RARE factor of 8, TR/effective TE 4200/36.3 ms (9.4 T) or 2500/42 ms (7 T), field of view 2×1.5 cm (9.4 T) or 2×2 cm (7 T), 12 coronal slices with a slice thickness of 0.5 mm, in plane resolution of 100 µm and a scan time of 1.5 min. For 3D T2-weighted brain scans, parameters were: RARE factor 10 (9.4 T) or 16 (7 T), TR/TE 1000/36 ms (9.4 T) or 1000/67 ms (7 T), FOV 2.4×1.5×0.83 cm, spatial resolution of approximately 94 µm in all directions and a scan time of 15 min.

### *Ex vivo* BLI and fungal load quantification

After the last *in vivo* BLI session, animals were sacrificed by i.p. injection of a pentobarbital overdose (Dolethal, Vetoquinol, Aartselaar, Belgium). Lungs, brains, spleens and kidneys of the animals were aseptically removed and placed in a 6-well plate inside the IVIS Spectrum system for *ex vivo* BLI. Images were acquired using an F/stop of 1 and medium binning, with exposure times depending on the magnitude of the signal (range 4-60 s). The weighted organs were homogenized in PBS and serial dilutions were plated on Sabouraud agar, incubated as specified above. All reported CFU counts represent the average of triplicate plating.

### Quantification of imaging results

#### Quantification of BLI results

Living Image software (version 4.5.4, Perkin Elmer, Waltham, USA) was used to quantify the total photon flux (photons/second) in the ROI. For *in vivo* BLI, the total photon flux in the lung, brain, nose or abdominal region was determined by placing a ROI over these regions with a size of 2.5×2.1 cm, 1.6×1.3 cm, 0.85×0.60 cm or 2.5×1.3 cm, respectively (Fig. S7). Consecutive frames with an exposure time of 1 min each were acquired for a period of 20 min. For each animal, the frame giving the highest total photon flux for the specific region was used for reporting.

For *ex vivo* BLI, the total photon flux in the isolated organs was measured by using a circular ROI for the lungs or brains (diameter 2.4 cm), both kidneys (1.4 cm diameter) or a rectangular ROI for the spleen (1.7×1.2 cm), respectively. For the lung and brain, three frames were acquired on both sides of the organ and averaged. To correct for scattering of strong bioluminescence from other organs in the well plate, the brain bioluminescence was expressed as the logarithm of the ratio of brain bioluminescence to background bioluminescence (identically sized ROI). For the spleen and kidneys, one frame was acquired.

#### Quantification of lung μCT data

Quantification was performed similarly to a previously described protocol (Vande Velde et al., 2016). All software was provided by the scanner manufacturer (Bruker micro-CT, Kontich, Belgium). The acquired lung scans were sorted to different phases in the breathing cycle (TSort, version 1.2.01) to reduce breathing artifacts, as previously described (De Langhe et al., 2012). Images were reconstructed by using the NRecon software (version 1.6.10.4) with the following parameters: Gaussian smoothing of 1%, beam hardening correction of 10%, and individual optimization of post alignment settings and ring artifact reduction (3% for all scans). We used an automated segmentation algorithm to quantify the connected aerated lung tissue for all four phases of the breathing cycle, as described in De Langhe et al. (2012) with minor adaptations. The dataset from the phase of the breathing cycle that corresponded to the end-expiratory volumes was selected for further data processing. ROIs covering the lungs but excluding the heart and main blood vessels were manually drawn on selected coronal images using the interpolation tool in CTAn software (version 1.16.3). The resulting volume of interest (VOI) was converted to a binary dataset by setting a fixed threshold of 73 on a scale of 0-255 in the grayscale index histogram. This allowed for segmentation of the air in the lungs (aerated lung volume, threshold 0-73) and the total lung volume (0-255). The residual volume (threshold 74-255), referred to as the tissue-lesion volume, includes both the normal tissue in the lungs (e.g. interstitial tissue and vessels) and the additional tissue deposited by the pulmonary infection.

#### Quantification of brain MRI data

Brain lesions were manually delineated on 2D or 3D images using the adaptive brush tool in ITK-SNAP (version 3.4.0, available via itksnap.org; Yushkevich et al., 2006). This manual segmentation allows quantification of the total lesion volume on the 3D scans. On the segmented scans, the number of lesions was determined by using the 3D object counter tool in ImageJ (version 1.49, National Institute of Health, USA; Bolte and Cordelières, 2006; Schneider et al., 2012). For the figures, a ROI covering the brain was manually drawn in ITK-SNAP and the 2D brain MRIs were subsequently masked by using ImageJ.

### Statistical analysis

All data were analyzed using GraphPad Prism (version 5.04, GraphPad Software Inc., San Diego, CA, USA). A two-tailed *P*-value of <0.05 was considered statistically significant. Statistical analysis of BLI and CFU data was performed on the log-transformed values for total photon flux and CFU counts. A two-way ANOVA with Bonferroni post-test was performed to compare the *in vitro* BLI signal for every cell concentration to the average background signal. Cross-sectional studies were analyzed using an unpaired *t*-test or one-way ANOVA. For longitudinal studies with available baseline values, a one-way repeated measures (RM) ANOVA was performed. A two-way RM ANOVA was used when comparing multiple infection groups or when comparing to uninfected controls, thereby assuming that the latter values would remain constant during the whole study period. A Bonferroni post-test was used to compare individual time points to baseline or uninfected controls. In cases of time points with missing values due to animal death, the RM analysis was performed with inclusion of all animals up to the point where the dataset was complete. After that, the last time points were analyzed by including only the remaining animals. Correlations were analyzed using Pearson correlation coefficients.

## Supplementary Material

Supplementary information

## References

[DMM039123C1] Anderson D. A. and Sagha H. M. (1988). Persistence of infection in mice inoculated intranasally with *Cryptococcus neoformans*. *Mycopathologia* 104, 163-169. 10.1007/BF00437432 3070384

[DMM039123C2] Andreu N., Zelmer A. and Wiles S. (2011). Noninvasive biophotonic imaging for studies of infectious disease. *FEMS Microbiol. Rev.* 35, 360-394. 10.1111/j.1574-6976.2010.00252.x 20955395 PMC3084502

[DMM039123C3] Arras S. D. M., Chitty J. L., Blake K. L., Schulz B. L. and Fraser J. A. (2015). A genomic safe haven for mutant complementation in *Cryptococcus neoformans*. *PLoS ONE* 10, e0122916 10.1371/journal.pone.0122916 25856300 PMC4391909

[DMM039123C4] Aswendt M., Adamczak J., Couillard-Despres S., Hoehn M., Bailly C. and Gaensler K. (2013). Boosting bioluminescence neuroimaging: an optimized protocol for brain studies. *PLoS ONE* 8, e55662 10.1371/journal.pone.0055662 23405190 PMC3566035

[DMM039123C5] Avci P., Karimi M., Sadasivam M., Antunes-Melo W. C., Carrasco E. and Hamblin M. R. (2018). *In-vivo* monitoring of infectious diseases in living animals using bioluminescence imaging. *Virulence* 9, 28-63. 10.1080/21505594.2017.1371897 28960132 PMC6067836

[DMM039123C6] Ayzenberg I., Schlevogt S., Metzdorf J., Stahlke S., Pedreitturia X., Hunfeld A., Couillard-Despres S. and Kleiter I. (2015). Analysis of neurogenesis during experimental autoimmune encephalomyelitis reveals pitfalls of bioluminescence imaging. *PLoS ONE* 10, e0118550 10.1371/journal.pone.0118550 25780928 PMC4363373

[DMM039123C7] Berger F., Paulmurugan R., Bhaumik S. and Gambhir S. S. (2008). Uptake kinetics and biodistribution of 14C-D-luciferin--a radiolabeled substrate for the firefly luciferase catalyzed bioluminescence reaction: impact on bioluminescence based reporter gene imaging. *Eur. J. Nucl. Med. Mol. Imaging* 35, 2275-2285. 10.1007/s00259-008-0870-6 18661130 PMC4157642

[DMM039123C8] Binder U., Navarro-Mendoza M. I., Naschberger V., Bauer I., Nicolas F. E., Pallua J. D., Lass-Flörl C., Garre V., Binder U., Navarro-Mendoza M. I. et al. (2018). Generation of a *Mucor circinelloides* reporter strain—a promising new tool to study antifungal drug efficacy and mucormycosis. *Genes (Basel)* 9, 613 10.3390/genes9120613 PMC6315630 30544643

[DMM039123C9] Bolte S. and Cordelières F. P. (2006). A guided tour into subcellular colocalization analysis in light microscopy. *J. Microsc.* 224, 213-232. 10.1111/j.1365-2818.2006.01706.x 17210054

[DMM039123C10] Brock M. (2012). Application of bioluminescence imaging for in vivo monitoring of fungal infections. *Int. J. Microbiol.* 2012, 956794 10.1155/2012/956794 22121368 PMC3205719

[DMM039123C11] Brock M., Jouvion G., Droin-Bergère S., Dussurget O., Nicola M.-A. and Ibrahim-Granet O. (2008). Bioluminescent Aspergillus fumigatus, a new tool for drug efficiency testing and *in vivo* monitoring of invasive aspergillosis. *Appl. Environ. Microbiol.* 74, 7023-7035. 10.1128/AEM.01288-08 18820063 PMC2583481

[DMM039123C12] Capilla J., Maffei C. M. L., Clemons K. V., Sobel R. A. and Stevens D. A. (2006). Experimental systemic infection with *Cryptococcus neoformans var. grubii* and *Cryptococcus gattii* in normal and immunodeficient mice. *Med. Mycol.* 44, 601-610. 10.1080/13693780600810040 17071553

[DMM039123C13] Charlier C., Nielsen K., Daou S., Brigitte M., Chretien F. and Dromer F. (2009). Evidence of a role for monocytes in dissemination and brain invasion by *Cryptococcus neoformans*. *Infect. Immun.* 77, 120-127. 10.1128/IAI.01065-08 18936186 PMC2612285

[DMM039123C14] Contag C. H., Contag P. R., Mullins J. I., Spilman S. D., Stevenson D. K. and Benaron D. A. (1995). Photonic detection of bacterial pathogens in living hosts. *Mol. Microbiol.* 18, 593-603. 10.1111/j.1365-2958.1995.mmi_18040593.x 8817482

[DMM039123C15] De Langhe E., Vande Velde G., Hostens J., Himmelreich U., Nemery B., Luyten F. P., Vanoirbeek J. and Lories R. J. (2012). Quantification of lung fibrosis and emphysema in mice using automated micro-computed tomography. *PLoS ONE* 7, e43123 10.1371/journal.pone.0043123 22912805 PMC3418271

[DMM039123C16] Del Poeta M. and Casadevall A. (2012). Ten challenges on Cryptococcus and cryptococcosis. *Mycopathologia* 173, 303-310. 10.1007/s11046-011-9473-z 21948062 PMC4294698

[DMM039123C17] Donat S., Hasenberg M., Schäfer T., Ohlsen K., Gunzer M., Einsele H., Löffler J., Beilhack A. and Krappmann S. (2012). Surface display of *Gaussia princeps* luciferase allows sensitive fungal pathogen detection during cutaneous aspergillosis. *Virulence* 3, 51-61. 10.4161/viru.3.1.18799 22286700

[DMM039123C18] Dorsaz S., Coste A. T. and Sanglard D. (2017). Red-shifted firefly luciferase optimized for *Candida albicans in vivo* bioluminescence imaging. *Front. Microbiol.* 8, 1478 10.3389/fmicb.2017.01478 28824601 PMC5541039

[DMM039123C19] Doyle T. C., Nawotka K. A., Kawahara C. B., Francis K. P. and Contag P. R. (2006). Visualizing fungal infections in living mice using bioluminescent pathogenic *Candida albicans* strains transformed with the firefly luciferase gene. *Microb. Pathog.* 40, 82-90. 10.1016/j.micpath.2005.11.003 16426810

[DMM039123C20] Enjalbert B., Rachini A., Vediyappan G., Pietrella D., Spaccapelo R., Vecchiarelli A., Brown A. J. P. and d'Enfert C. (2009). A multifunctional, synthetic *Gaussia princeps* luciferase reporter for live imaging of *Candida albicans* infections. *Infect. Immun.* 77, 4847-4858. 10.1128/IAI.00223-09 19687206 PMC2772526

[DMM039123C21] Galiger C., Brock M., Jouvion G., Savers A., Parlato M. and Ibrahim-Granet O. (2013). Assessment of efficacy of antifungals against *Aspergillus fumigatus*: value of real-time bioluminescence imaging. *Antimicrob. Agents Chemother.* 57, 3046-3059. 10.1128/AAC.01660-12 23587947 PMC3697358

[DMM039123C22] Goebels C., Thonn A., Gonzalez-Hilarion S., Rolland O., Moyrand F., Beilharz T. H. and Janbon G. (2013). Introns regulate gene expression in *Cryptococcus neoformans* in a Pab2p dependent pathway. *PLoS Genet.* 9, e1003686 10.1371/journal.pgen.1003686 23966870 PMC3744415

[DMM039123C23] He X., Lyons D. M., Toffaletti D. L., Wang F., Qiu Y., Davis M. J., Meister D. L., Dayrit J. K., Lee A., Osterholzer J. J. et al. (2012). Virulence factors identified by *Cryptococcus neoformans* mutant screen differentially modulate lung immune responses and brain dissemination. *Am. J. Pathol.* 181, 1356-1366. 10.1016/j.ajpath.2012.06.012 22846723 PMC3463625

[DMM039123C24] Himmelreich U., Sorrell T., Dzendrowskyj T. E., Malik R. and Mountford C. (2002). Identification of *Cryptococcus neoformans* by Magnetic Resonance Spectroscopy. *Microbiol. Aust.* 23, 31-33.

[DMM039123C25] Hutchens M. and Luker G. D. (2007). Applications of bioluminescence imaging to the study of infectious diseases. *Cell. Microbiol.* 9, 2315-2322. 10.1111/j.1462-5822.2007.00995.x 17587328

[DMM039123C26] Ibrahim-Granet O., Jouvion G., Hohl T. M., Droin-Bergère S., Philippart F., Kim O. Y., Adib-Conquy M., Schwendener R., Cavaillon J.-M. and Brock M. (2010). In vivo bioluminescence imaging and histopathopathologic analysis reveal distinct roles for resident and recruited immune effector cells in defense against invasive aspergillosis. *BMC Microbiol.* 10, 105 10.1186/1471-2180-10-105 20377900 PMC2859869

[DMM039123C27] Jacobsen I. D., Lüttich A., Kurzai O., Hube B. and Brock M. (2014). In vivo imaging of disseminated murine *Candida albicans* infection reveals unexpected host sites of fungal persistence during antifungal therapy. *J. Antimicrob. Chemother.* 69, 2785-2796. 10.1093/jac/dku198 24951534

[DMM039123C28] Kaufman-Francis K., Djordjevic J. T., Juillard P.-G., Lev S., Desmarini D., Grau G. E. R. and Sorrell T. C. (2018). The early innate immune response to, and phagocyte-dependent entry of, *Cryptococcus neoformans* map to the perivascular space of cortical post-capillary venules in neurocryptococcosis. *Am. J. Pathol.* 188, 1653-1665. 10.1016/j.ajpath.2018.03.015 29929915

[DMM039123C29] Lakatos H. F., Burgess H. A., Thatcher T. H., Redonnet M. R., Hernady E., Williams J. P. and Sime P. J. (2006). Oropharyngeal aspiration of a silica suspension produces a superior model of silicosis in the mouse when compared to intratracheal instillation. *Exp. Lung Res.* 32, 181-199. 10.1080/01902140600817465 16908446 PMC10208218

[DMM039123C30] Lin X. and Heitman J. (2006). The biology of the *Cryptococcus neoformans* species complex. *Annu. Rev. Microbiol.* 60, 69-105. 10.1146/annurev.micro.60.080805.142102 16704346

[DMM039123C31] Liu T.-B., Perlin D. S. and Xue C. (2012). Molecular mechanisms of cryptococcal meningitis. *Virulence* 3, 173-181. 10.4161/viru.18685 22460646 PMC3396696

[DMM039123C32] Mosci P., Pericolini E., Gabrielli E., Kenno S., Perito S., Bistoni F., d'Enfert C. and Vecchiarelli A. (2013). A novel bioluminescence mouse model for monitoring oropharyngeal candidiasis in mice. *Virulence* 4, 250-254. 10.4161/viru.23529 23334179 PMC3711983

[DMM039123C33] Muhammed M., Feldmesser M., Shubitz L. F., Lionakis M. S., Sil A., Wang Y., Glavis-Bloom J., Lewis R. E., Galgiani J. N., Casadevall A. et al. (2012). Mouse models for the study of fungal pneumonia: a collection of detailed experimental protocols for the study of *Coccidioides, Cryptococcus, Fusarium, Histoplasma* and combined infection due to *Aspergillus-Rhizopus*. *Virulence* 3, 329-338. 10.4161/viru.20142 22546902 PMC3442846

[DMM039123C34] Ngamskulrungroj P., Chang Y., Sionov E. and Kwon-Chung K. J. (2012). The primary target organ of Cryptococcus gattii is different from that of Cryptococcus neoformans in a murine model. *MBio* 3, e00103-e00112. 10.1128/mBio.00103-12 22570277 PMC3350374

[DMM039123C35] Pai M. P., Sakoglu U., Peterson S. L., Lyons C. R. and Sood R. (2009). Characterization of BBB permeability in a preclinical model of cryptococcal meningoencephalitis using magnetic resonance imaging. *J. Cereb. Blood Flow Metab.* 29, 545-553. 10.1038/jcbfm.2008.144 19066614

[DMM039123C36] Poelmans J., Hillen A., Vanherp L., Govaerts K., Maertens J., Dresselaers T., Himmelreich U., Lagrou K. and Vande Velde G. (2016). Longitudinal, *in vivo* assessment of invasive pulmonary aspergillosis in mice by computed tomography and magnetic resonance imaging. *Lab. Invest.* 96, 692-704. 10.1038/labinvest.2016.45 27019389

[DMM039123C37] Poelmans J., Himmelreich U., Vanherp L., Zhai L., Hillen A., Holvoet B., Belderbos S., Brock M., Maertens J., Vande Velde G. et al. (2018). A multimodal imaging approach enables *in vivo* assessment of antifungal treatment in a mouse model of invasive pulmonary aspergillosis. *Antimicrob. Agents Chemother* 62, AAC.00240-18 10.1128/AAC.00240-18 PMC6021662 29760132

[DMM039123C38] Santry L. A., Ingrao J. C., Yu D. L., de Jong J. G., van Lieshout L. P., Wood G. A. and Wootton S. K. (2017). AAV vector distribution in the mouse respiratory tract following four different methods of administration. *BMC Biotechnol.* 17, 43 10.1186/s12896-017-0365-2 28506256 PMC5433059

[DMM039123C39] Schneider C. A., Rasband W. S. and Eliceiri K. W. (2012). NIH Image to ImageJ: 25 years of image analysis. *Nat. Methods* 9, 671-675. 10.1038/nmeth.2089 22930834 PMC5554542

[DMM039123C40] Schwartz S., Kontoyiannis D. P., Harrison T. and Ruhnke M. (2018). Advances in the diagnosis and treatment of fungal infections of the CNS. *Lancet Neurol.* 17, 362-372. 10.1016/S1474-4422(18)30030-9 29477506

[DMM039123C41] Shi M., Li S. S., Zheng C., Jones G. J., Kim K. S., Zhou H., Kubes P. and Mody C. H. (2010). Real-time imaging of trapping and urease-dependent transmigration of Cryptococcus neoformans in mouse brain. *J. Clin. Invest.* 120, 1683-1693. 10.1172/JCI41963 20424328 PMC2860939

[DMM039123C42] Tenor J. L., Oehlers S. H., Yang J. L., Tobin D. M. and Perfect J. R. (2015). Live imaging of host-parasite interactions in a zebrafish infection model reveals cryptococcal determinants of virulence and central nervous system invasion. *MBio* 6, e01425-15 10.1128/mBio.01425-15 26419880 PMC4611042

[DMM039123C43] Toffaletti D. L., Rude T. H., Johnston S. A., Durack D. T. and Perfect J. R. (1993). Gene transfer in *Cryptococcus neoformans* by use of biolistic delivery of DNA. *J. Bacteriol.* 175, 1405-1411. 10.1128/jb.175.5.1405-1411.1993 8444802 PMC193227

[DMM039123C44] Vande Velde G. and Wiehr S. (2017). Fungal imaging. In *Imaging Infections* (ed. Jain S. K.), pp. 173-183. Springer.

[DMM039123C45] Vande Velde G., Kucharíková S., Schrevens S., Himmelreich U. and Van Dijck P. (2014). Towards non-invasive monitoring of pathogen-host interactions during Candida albicans biofilm formation using in vivo bioluminescence. *Cell. Microbiol.* 16, 115-130. 10.1111/cmi.12184 23962311 PMC4204156

[DMM039123C46] Vande Velde G., Poelmans J., De Langhe E., Hillen A., Vanoirbeek J., Himmelreich U. and Lories R. J. (2016). Longitudinal micro-CT provides biomarkers of lung disease that can be used to assess the effect of therapy in preclinical mouse models, and reveal compensatory changes in lung volume. *Dis. Model. Mech.* 9, 91-98. 10.1242/dmm.020321 26563390 PMC4728330

[DMM039123C47] Vande Velde G., Kucharíková S., Van Dijck P. and Himmelreich U. (2018). Bioluminescence imaging increases in vivo screening efficiency for antifungal activity against device-associated Candida albicans biofilms. *Int. J. Antimicrob. Agents* 52, 42-51. 10.1016/j.ijantimicag.2018.03.007 29572043

[DMM039123C48] Vanherp L., Poelmans J., Hillen A., Govaerts K., Belderbos S., Buelens T., Lagrou K., Himmelreich U. and Vande Velde G. (2018). Bronchoscopic fibered confocal fluorescence microscopy for longitudinal *in vivo* assessment of pulmonary fungal infections in free-breathing mice. *Sci. Rep.* 8, 3009 10.1038/s41598-018-20545-4 29445211 PMC5813038

[DMM039123C49] Warawa J. M., Long D., Rosenke R., Gardner D. and Gherardini F. C. (2011). Bioluminescent diagnostic imaging to characterize altered respiratory tract colonization by the *burkholderia pseudomallei* capsule mutant. *Front. Microbiol.* 2, 133 10.3389/fmicb.2011.00133 21720539 PMC3118415

[DMM039123C50] Yushkevich P. A., Piven J., Hazlett H. C., Smith R. G., Ho S., Gee J. C. and Gerig G. (2006). User-guided 3D active contour segmentation of anatomical structures: significantly improved efficiency and reliability. *Neuroimage* 31, 1116-1128. 10.1016/j.neuroimage.2006.01.015 16545965

