# Supplementary material for: Sensitive bioluminescence imaging of fungal dissemination to the brain in mouse models of cryptococcosis
Source: Dis Model Mech. 2019 Jun 17;12(6):dmm039123. doi: 10.1242/dmm.039123 (PMC6602310; doi:10.1242/dmm.039123)
Supplement: Supplementary information [file dmm-12-039123-s1.pdf]

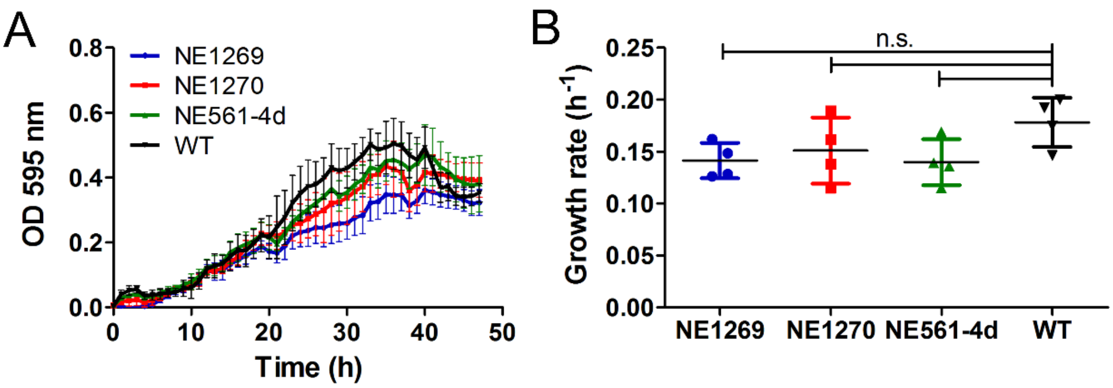

Figure S1. Integration of the luciferase-construct did not alter the growth rate of the strain. The optical density (OD, 595 nm) of the wild-type (WT) *C. neoformans* KN99α strain and the three transformants in Sabouraud medium was measured for 48 hours. A) The growth of the strains was comparable. B) The growth rates were quantified by fitting a logistic growth model (up to 40 hours). No significant differences were observed. Graphs show mean ± s.d.; n.s.: not significant, one-way ANOVA with Bonferroni post-test.

Disease Models & Mechanisms • Supplementary information

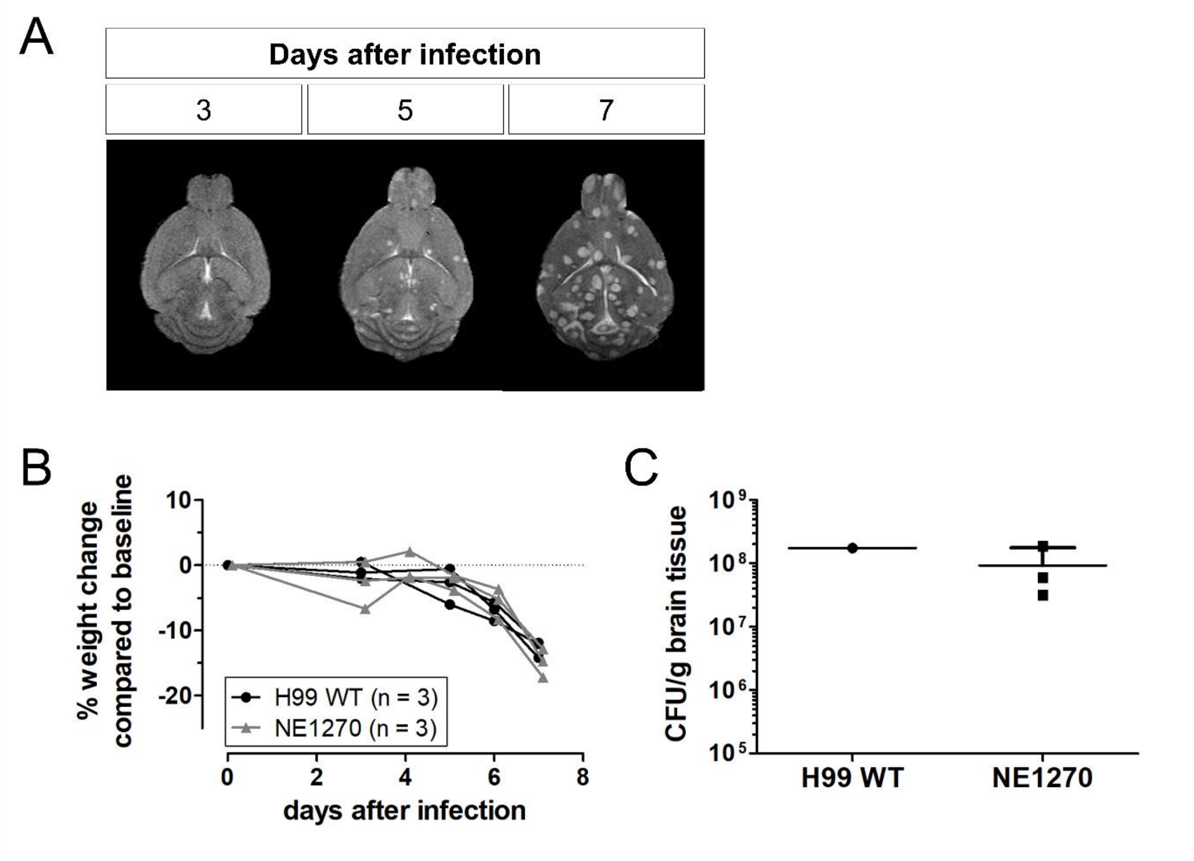

Figure S2. Intravenous infection with wild-type H99 caused similar brain pathology as with the bioluminescent NE1270 strain. A) MRI showed formation of multiple lesions in the brain after intravenous injection of 50,000 *C. neoformans* H99 cells, as was observed with the NE1270 strain (Fig. 2). B) The decrease in body weight of infected animals was similar for wild-type H99 (n = 3) and the bioluminescent NE1270 strain (n = 3). C) The fungal load in the brain was comparable for both strains (H99, n = 1 and NE1270, n = 3). Graphs show individual values or mean + s.d.

Disease Models & Mechanisms • Supplementary information

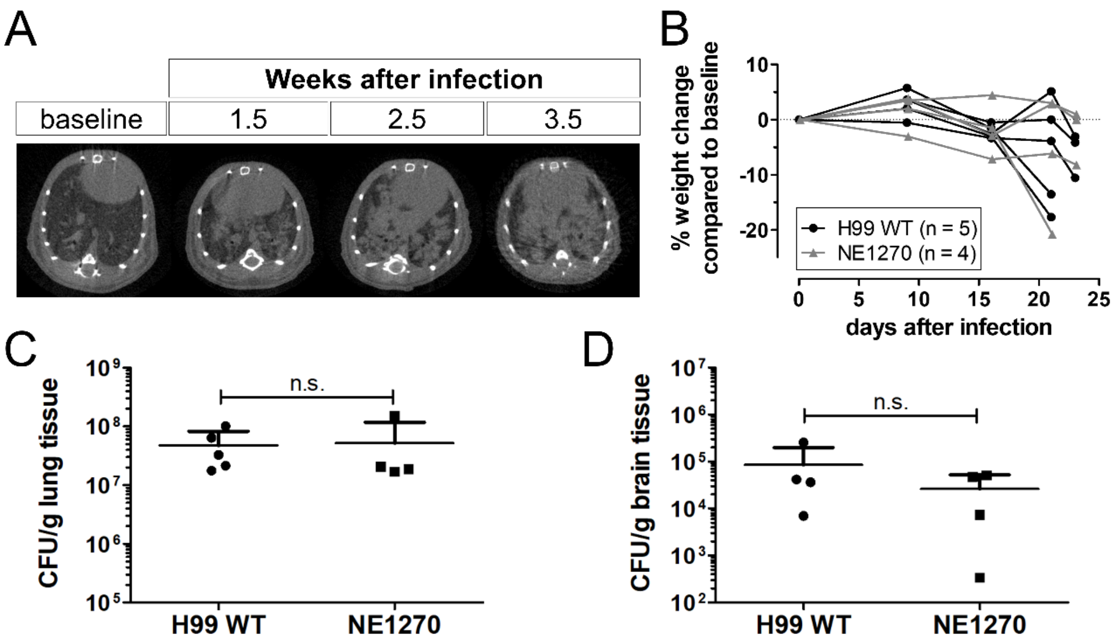

Figure S3. Intranasal infection with wild-type H99 or the bioluminescent NE1270 strain causes similar lung and brain pathology. A) CT showed development of pulmonary lesions after intranasal infection with 50,000 *C. neoformans* H99 cells, comparable to the NE1270 strain (Fig. 3). B) Changes in mouse body weight after infection were similar for infection with both the H99 WT and NE1270 strains. C) The fungal load in the lungs at the end stage of disease (21-23 days) was comparable for both strains (p = 0.7565). D) Similarly, no significant differences were observed in the fungal load in the brain (p = 0.3120). n.s.: not significant, unpaired t-test. Graphs show individual values or mean + s.d.

Disease Models & Mechanisms • Supplementary information

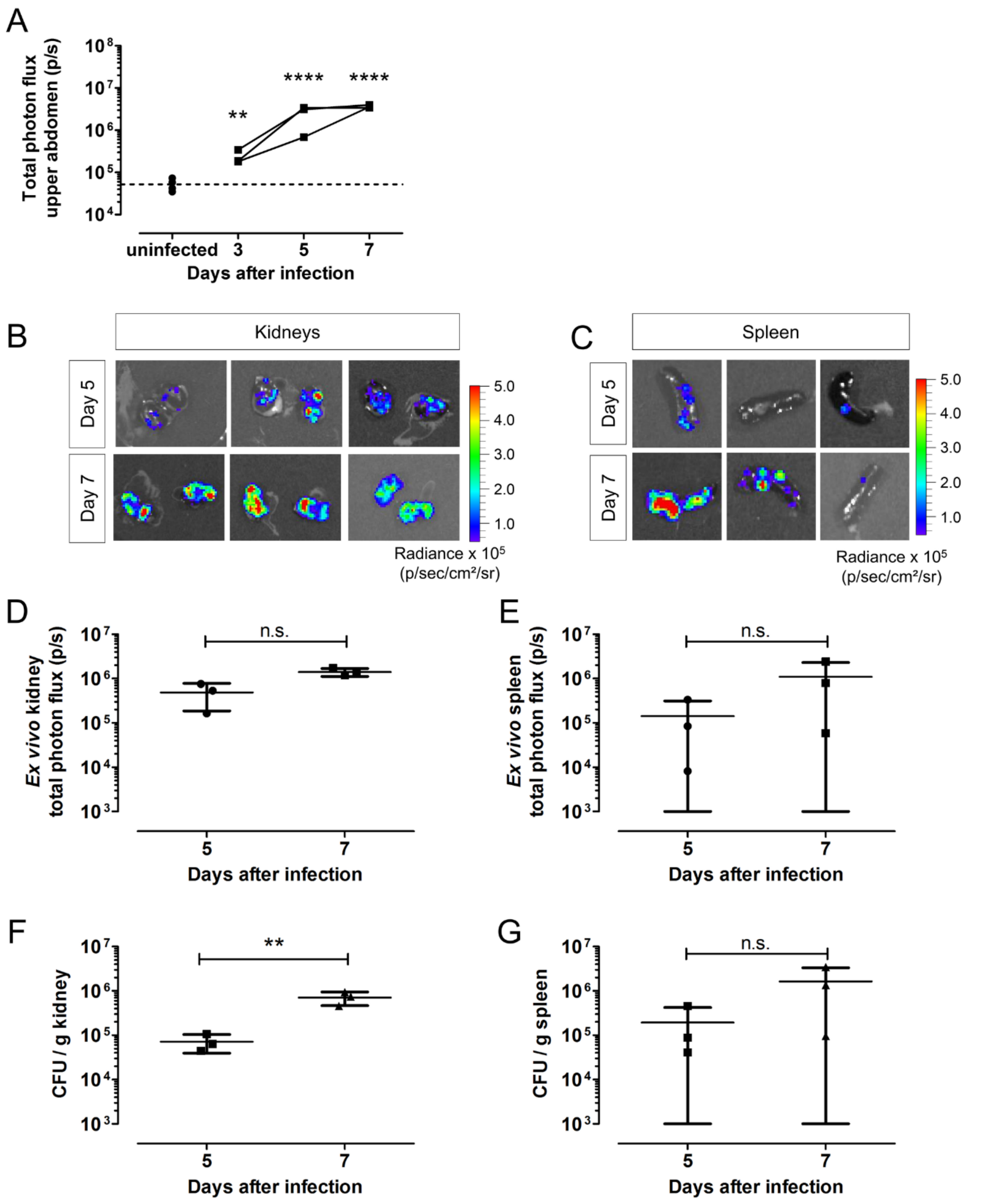

Figure S4. BLI showed dissemination to the spleen and kidney upon systemic infection. A) Mice (n = 3) were infected via intravenous injection of 50,000 *C. neoformans* KN99α-CnFLuc cells and scanned longitudinally using BLI until day 7, showing an increase in the bioluminescent signal intensity with time. B-G) At

day 7, *ex vivo* BLI and CFU counting of isolated spleen and kidneys was performed. Additional animals (n = 3) were included to obtain *ex vivo* data at day 5. Strong *ex vivo* bioluminescence was detected from the kidneys (B, D) and spleens (C,E) of infected animals. F-G) CFU analysis confirmed the presence of fungal cells in both organs. Graphs show the values for individual animals with mean and s.d. **: $p<0.01$; ****: $p<0.0001$; t-test or RM one-way ANOVA with Bonferroni post-test.

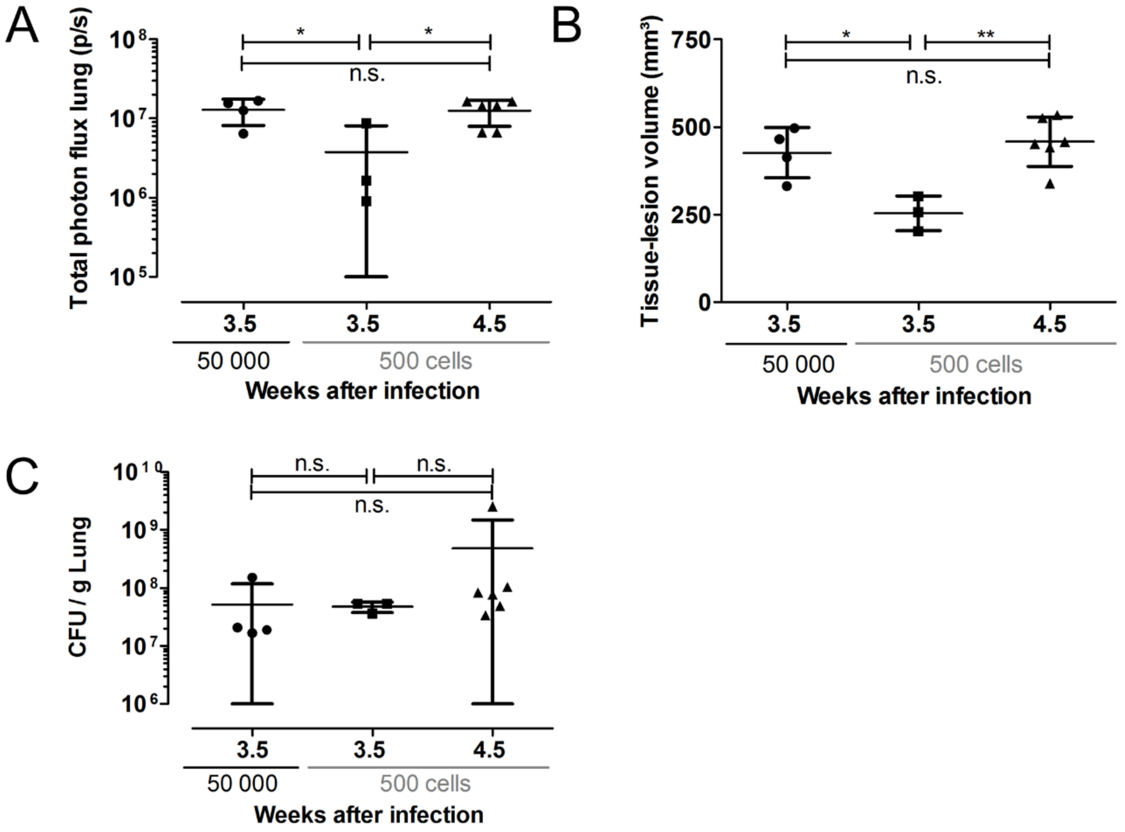

Figure S5. BLI and CT can differentiate the extent of pulmonary infection upon high and low inocula, unlike CFU counts. BLI (A) and CT (B) discriminated between the extent of infection in both groups, while CFU analysis (C) showed no (significant) differences. Data included the last time point from longitudinal studies, week 3.5 for animals infected intranasally with 50,000 cells (n = 4) or week 4.5 for 500 cells (n = 3). For the inoculum of 500 cells, additional animals from cross-sectional studies were included on week 3.5 and 4.5 (n = 3 each). Graphs show values for individual animals with mean and s.d. *: $p<0.05$, one-way ANOVA with Bonferroni post-test.

Disease Models & Mechanisms • Supplementary information

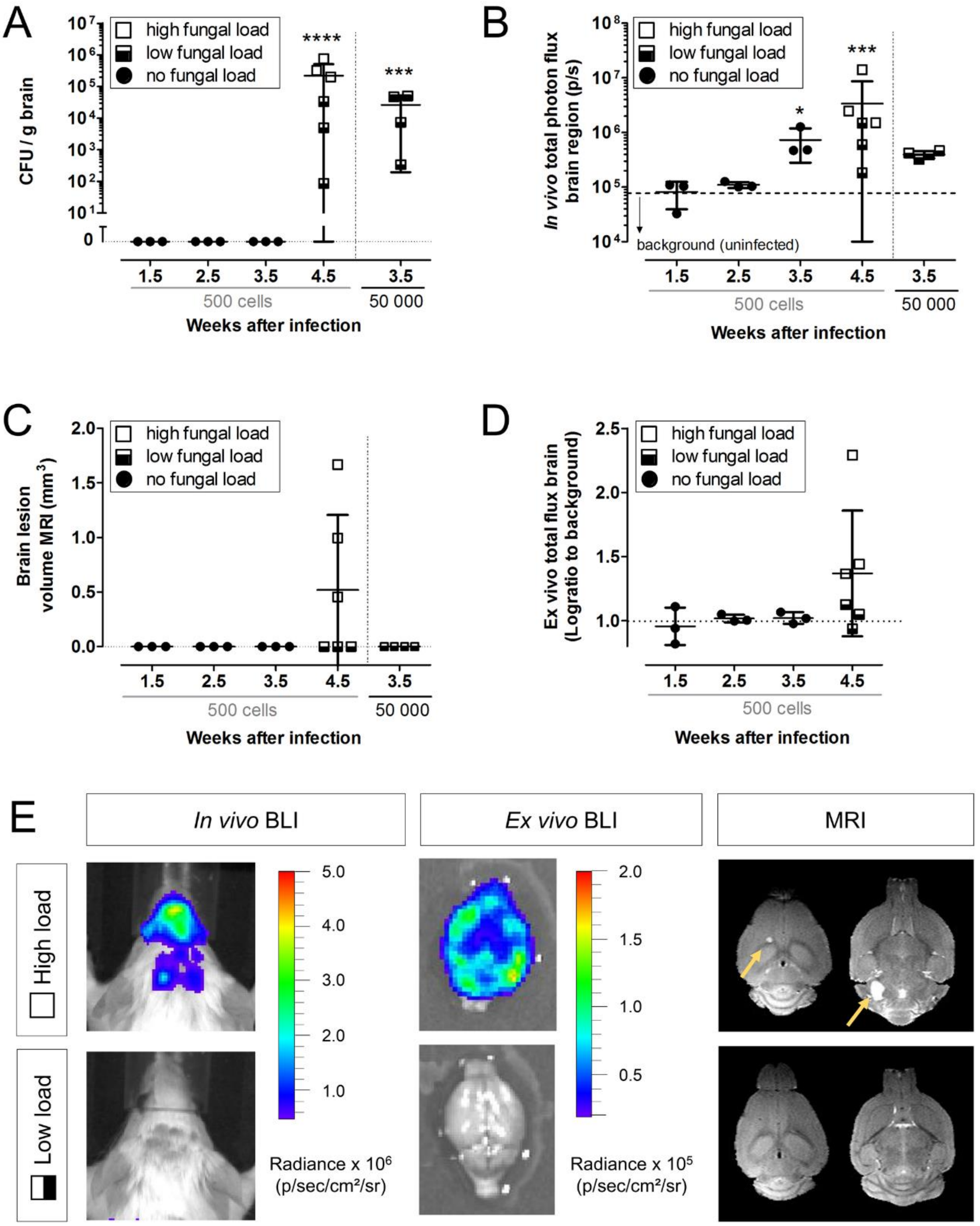

Figure S6. Cross-validation of CFU counts, *in vivo* BLI and MRI and *ex vivo* BLI as read-outs of brain disease in the intranasal model. The end-point measurements from the longitudinal studies (at 4.5 weeks in the 500 cells inoculum, n = 3, or 3.5 weeks in the 50,000 cells inoculum, n = 4) were combined with

Disease Models & Mechanisms • Supplementary information

data from a cross-sectional study whereby every week a group of animals (n = 3), infected with 500 cells, was imaged once by BLI and brain MRI. Subsequently, animals were sacrificed for *ex vivo* BLI and CFU counting of the brain. A) CFU counting detected fungal cells in the brain at week 4.5 (500 cells inoculum) or 3.5 p.i. (50,000 cells inoculum). Based on this, animals were classified in groups of high (> 10^5 CFUs/ gram brain), low or no fungal load. B) The *in vivo* bioluminescence from the brain region increased significantly above background from 3.5 weeks onwards (500 cells inoculum), although no CFUs could be retrieved at this point. At week 4.5 (500 cells inoculum) and 3.5 (50,000 cells inoculum), the detected total photon flux was highest for the animals with a high fungal load. C-D) The high fungal load group showed lesions on MRI (C) and *ex vivo* bioluminescence signals higher than background (D), while neither could be observed in the low fungal load group. E) Animals with high fungal load in the brain (top row) presented with high *in vivo* bioluminescence signal in the brain region and signs of brain involvement on *ex vivo* BLI and MRI (2 representative slices of the 2D scan of the same animal). In animals with a lower fungal brain burden (bottom row), the presence of infection could not be confirmed by *ex vivo* BLI or MRI. Graphs show scatter plots with mean and s.d. *: $p<0.05$; ***: $p<0.001$; ****: $p<0.0001$, one-way ANOVA with Bonferroni post-test, in comparison to uninfected controls (*in vivo* BLI, n = 4) or 1.5 weeks p.i. (CFU, MRI and *ex vivo* BLI).

Disease Models & Mechanisms • Supplementary information

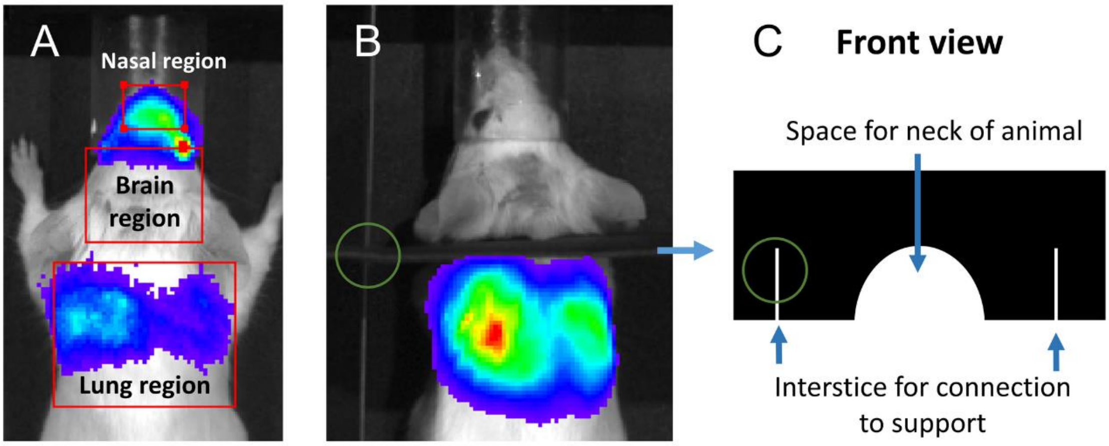

Figure S7. Methodological details for BLI. A) Placement of the regions of interest (ROIs) for quantification of the total photon flux in the nasal, brain or lung region. B) To separate the bioluminescence of the brain region from the lung region, a black partition was placed over the neck of the animal. C) Illustration of the design of the partition. Via the interstices, the partition can be connected to the separators placed between different animals.

Disease Models & Mechanisms • Supplementary information

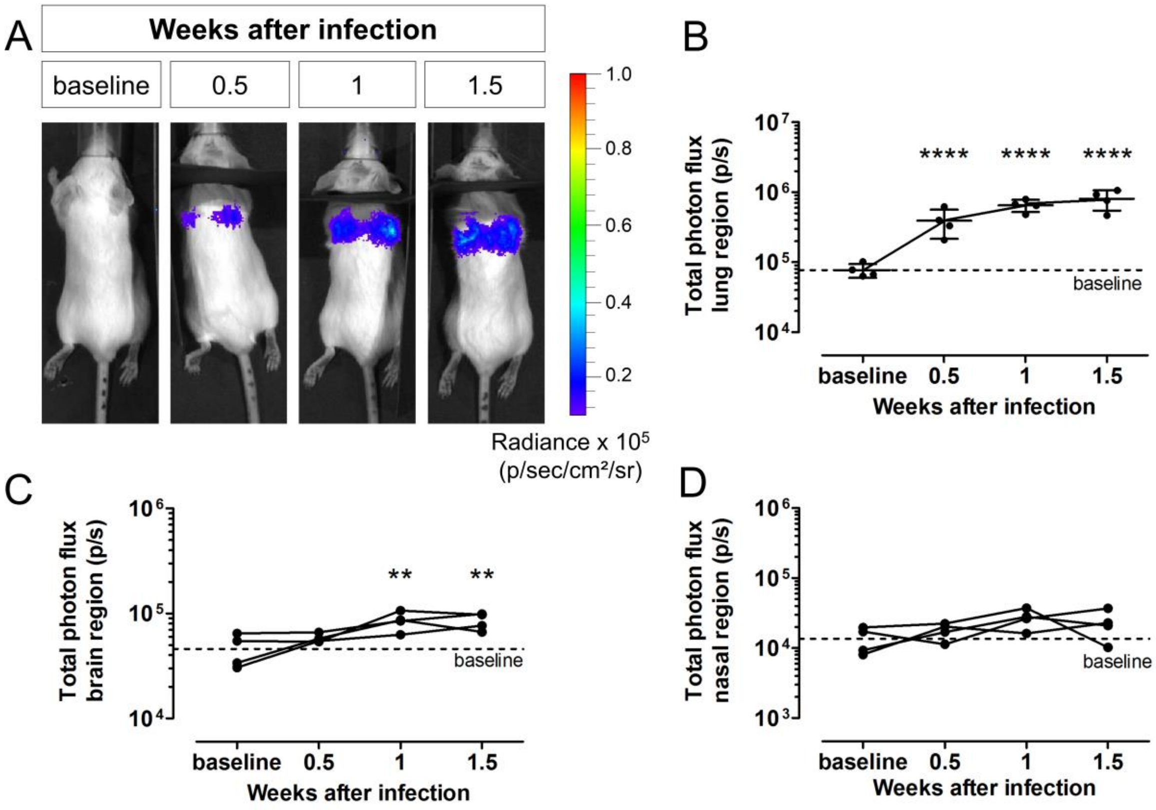

Figure S8. BLI of animals infected endotracheally with 50,000 bioluminescent *C. neoformans* KN99α cells. A-B) BLI showed the rapid development of a progressive pulmonary infection (n = 4). C) The bioluminescence from the brain region increased mildly. CFU analysis showed 0 to maximum 27 CFUs per gram brain tissue. D) No significant signal from the nasal area of the animals was observed. Data are represented as dots for individual animals with mean and s.d., or as connected lines for individual subjects. **: $p<0.01$; ****: $p<0.0001$; RM one-way ANOVA with Bonferroni post-test.

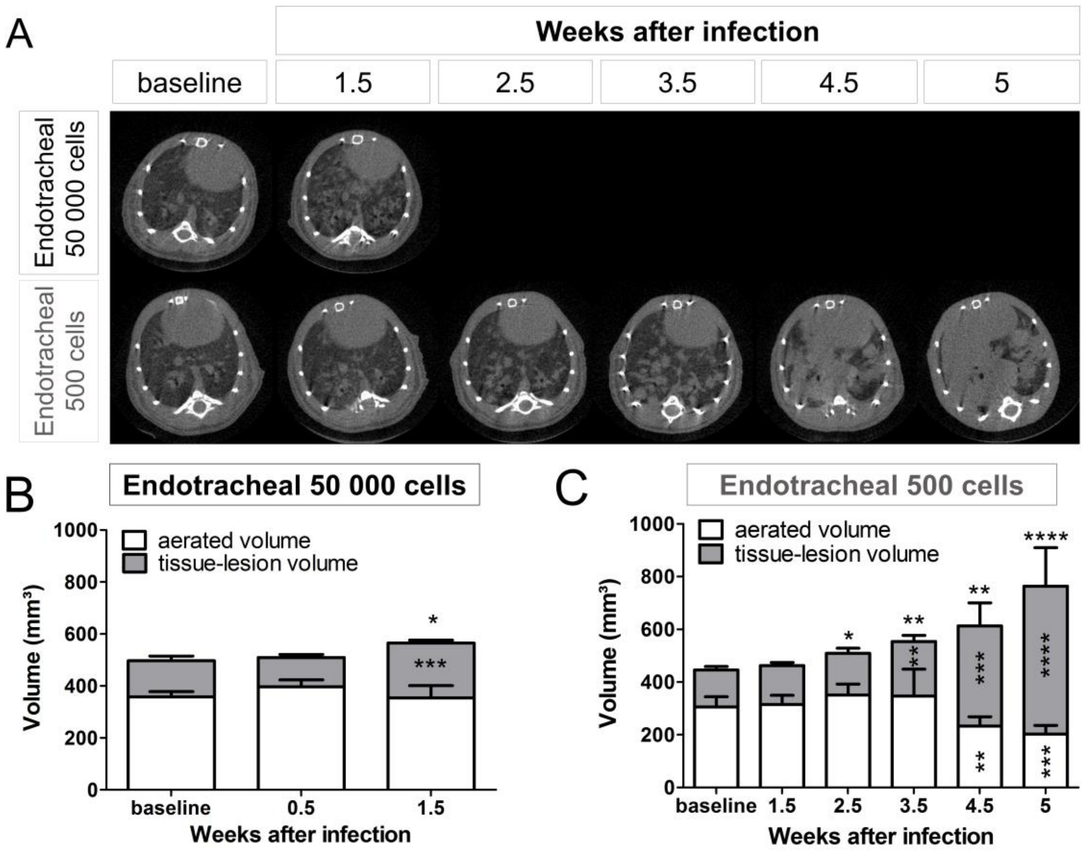

Figure S9. CT of the lungs in the endotracheal model. Mice were infected with 50,000 (n = 4) or 500 *C. neoformans* KN99α-CnFLuc cells via the endotracheal route (n = 12). From the latter group, 4 animals were excluded due to no or only slow infection development, and 2 additional animals did not have sufficient quality of the CT data at week 3.5, leaving 6 animals in the final dataset. A) Infected animals developed dense tissue patches (grey) in the normally aerated lung tissue (black). B and C) As for the intranasal model, the tissue-lesion volume increased and aerated lung volume decreased in the high- (B) and low- (C) inoculum groups, resulting in an increased total lung volume. Graphs represent mean with s.d. *: p<0.05; **: p<0.01; ***: p<0.001; ****: p<0.0001; RM one-way ANOVA with Bonferroni post-test. Significance for total lung volume is indicated on top of the bars.

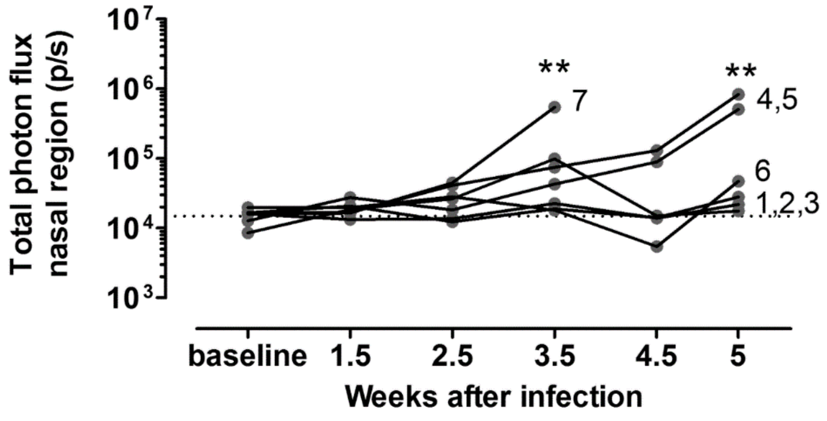

Figure S10. Bioluminescence in the nasal region after endotracheal inoculation (500 cells). For most animals, the signal from the nasal region was absent. Some animals (4, 5, 7) showed bioluminescence signals above baseline values. Graph shows data from individual animals. **: $p<0.01$; RM one-way ANOVA with Bonferroni post-test.

Disease Models & Mechanisms • Supplementary information

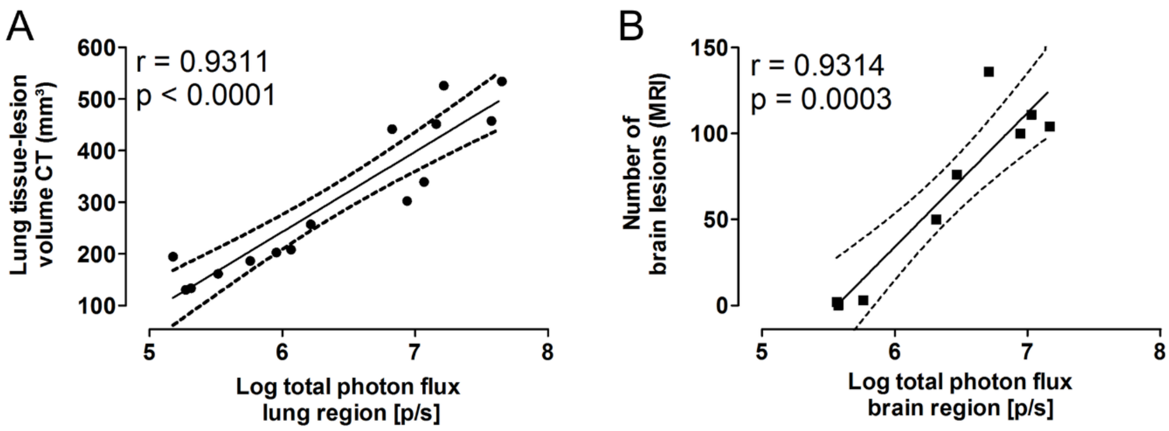

Figure S11. Cross-correlation of BLI and anatomical imaging (CT/ MRI) in the intranasal and intravenous model. A) Mice were infected intranasally with 500 *C. neoformans* KN99α-CnFLuc cells and subsequently scanned with BLI and lung CT at 1.5, 2.5, 3.5 (n = 3 per time point) or 4.5 weeks (n = 6, including longitudinally scanned animals) after infection. *In vivo* BLI of the lung correlates with the tissue lesion volume in the lungs. B) Animals infected intravenously with 50,000 *C. neoformans* cells were scanned using BLI and MRI on day 3, 5 or 7 (n = 3 each). The *in vivo* BLI signal correlates strongly with the number of brain lesions seen on MRI. Graphs show individual data points, Pearson correlation coefficient and regression lines with 95% confidence bands.
